# Prediction of RNA Pseudoknots Using Heuristic Modeling with Mapping and Sequential Folding

**DOI:** 10.1371/journal.pone.0000905

**Published:** 2007-09-19

**Authors:** Wayne K. Dawson, Kazuya Fujiwara, Gota Kawai

**Affiliations:** Department of Life and Environmental Sciences, Chiba Institute of Technology, Narashino-shi, Chiba, Japan; Vanderbilt University, United States of America

## Abstract

Predicting RNA secondary structure is often the first step to determining the structure of RNA. Prediction approaches have historically avoided searching for pseudoknots because of the extreme combinatorial and time complexity of the problem. Yet neglecting pseudoknots limits the utility of such approaches. Here, an algorithm utilizing structure mapping and thermodynamics is introduced for RNA pseudoknot prediction that finds the minimum free energy and identifies information about the flexibility of the RNA. The heuristic approach takes advantage of the 5′ to 3′ folding direction of many biological RNA molecules and is consistent with the hierarchical folding hypothesis and the contact order model. Mapping methods are used to build and analyze the folded structure for pseudoknots and to add important 3D structural considerations. The program can predict some well known pseudoknot structures correctly. The results of this study suggest that many functional RNA sequences are optimized for proper folding. They also suggest directions we can proceed in the future to achieve even better results.

## Introduction

A large percentage of RNA in the cell is composed of RNA that folds up into complex structures [1], [2] that are often described in terms of their base pairing configurations known as secondary structure (See Text S1 for an introduction to this topic). RNA pseudoknots are a class of base pairing structures that appear in many viruses and may comprise as much as 10% of all RNA structures [3]. However, including the complete repertoire of pseudoknots in RNA structure prediction can drastically increase the demands on computational resources [4].

RNA structure studies provide important information about the mechanisms behind functional RNA. Viruses utilize pseudoknots for mimicry [5], [6] and frame shifting [7]–[9]. An understanding of RNA folding and dynamics is likely to speed up the discovery of critical targets that aid in the rapid development of vaccines in the case of a pandemic [10] and can help model the structure of unknown non-coding RNA sequences that comprises a large fraction of the human genome [11].

Currently, there are no approaches that consider the long-range effects of polymer-solvent interactions such as swelling and the formation of a globular state. Nor is persistence length (or Kuhn length) used to learn the flexibility (rigidity) of RNA structures: neither alone nor incorporated with swelling. Evolution shows a general selectivity for folding sequences [12], therefore, it should be able to select sequences that make use of this 5′ to 3′ folding process (during transcription) to insure that the native state is found efficiently. Modeling techniques should be able to take advantage of this feature of biological RNA.

To address the question of pseudoknots in RNA structure, we have expanded and further developed a program (vsfold4) that calculates secondary structure [13]. Expanding secondary structure methods to handle all pseudoknots typically costs far more computer resources [1], [4], [14]. Vsfold5 is a unique approach that makes it possible to transition directly to the pseudoknot (PK) problem. The worst case introduces at most a factor *N* to the computation time (known as time complexity), where *N* is the sequence length.

Here we present a unique pseudoknot modeling algorithm using thermodynamics that utilizes sequential (5′ to 3′) folding along the thermodynamically most-probable folding-pathway, permits use of more realistic polymer models that can handle globular conditions, can find optimal structures including pseudoknots on RNA sequences efficiently, and is unique in using mapping routines of pointers (for secondary structure) and handles (for pseudoknots) to build, parse, analyze and predict the RNA structure as it is folding. The results suggest that RNA-folding in the cell has evolved strategies that help promote the formation of correct structure. They further suggest the direction we need to pursue to achieve better thermodynamic models of RNA. Finally, polymer-solvent effects are predicted for the first time in computational programs of this kind.

## Results and Discussion

**Figure 1:**
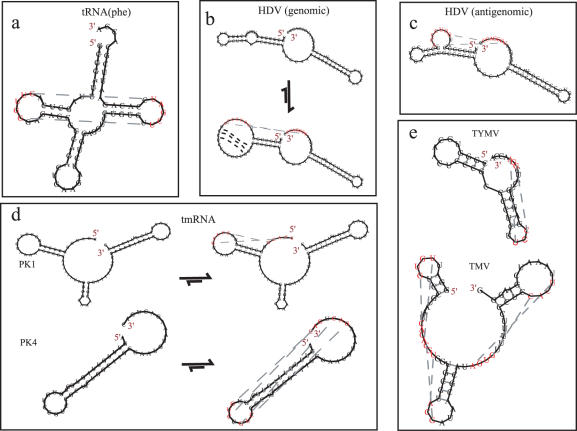
Examples of predicted pseudoknot structures. (a) An example of base pairing between the D–T loop regions of *E. coli* tRNA^(Phe)^–predicted with default parameters. (b,c) Prediction of the hepatitis delta virus (HDV) self cleavage ribozymes for the genomic (b) and antigenomic (c) sequences (Kuhn length 8 nt). For the genomic HDV (b), the secondary structure prediction is also shown above. The secondary structure of the antigenomic HDV is unchanged by pseudoknot formation. (d) Examples of predictions of the pseudoknots in *E. coli* tmRNA: (PK1) PK1 with default parameters, (PK4) PK4 with Kuhn length of 7 nt and minimum stem length 3 bp. On the left is the predicted secondary structure alone, and on the right, the same prediction including the pseudoknot option. (e) The turnip yellow mosaic virus (TYMV) and the tandem pseudoknots of the tobacco mosaic virus (TMV) frame shift sequence. Structures created using a modified version of naview [15].

**Figure 2:**
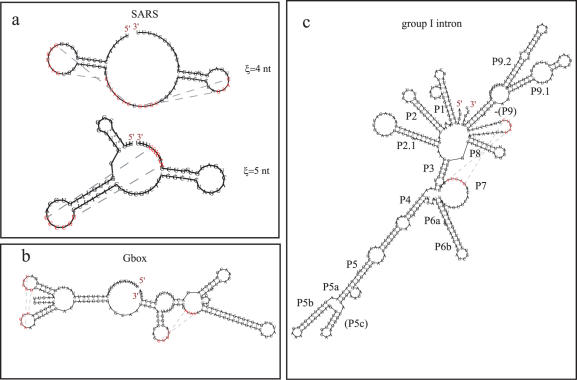
Other examples of pseudoknot predictions. (a) The frame shift sequence of the SARS corona virus for two different Kuhn lengths: *ξ* = 4 nt and *ξ* = 5 nt. The measured structure is closer to *ξ* = 5 nt. (b) G-box structure predicted for the full sequence: Kuhn length *ξ* = 4 to 5 nt with the effective Flory option, *δ*∼1.6, and other parameters default. (c) *T. thermophila* Group I intron structure predicted for the full sequence: *ξ* = 10 nt, using effective Flory with settings *δ* = 1.4 and *γ* = 1.5; where ‘P’ indicates known pairing, (..) indicate some prediction errors, and ‘-(..)‘ indicates missed in the prediction.

For a number of familiar pseudoknots, the 5′ to 3′ sequential folding appears to capture the general features of the pseudoknot structure successfully (Figs. 1 and 2).

### tRNA tertiary structure

When tRNA has sufficiently many contiguous base pairs located around the D-T loop overlap, a pseudoknot is predicted for *E. coli* tRNA^(Phe)^—Fig. 1a.

### Human delta virus (HDV)

For the self cleavage ribozyme in the human delta virus (HDV), both the genomic form and the antigenomic form are captured in the pseudoknot structure (Figs. 1b and c) [16]. The predicted secondary structure is also shown for reference. The genomic form (Fig. 1b) of the structure largely forms by melting in the leading edge onto the first domain. Some structure is lost in the process first because of an assumed fixed Kuhn length of 8 nucleotides (nt) and second because there is currently no stability enhancement introduced by long range coaxial stacking effects on this short stem. (The Kuhn length measures the rigidity of the RNA and therefore tends to favorably weight the distribution of stem lengths accordingly.) Nevertheless, the most significant parts of the structure are found.

### tmRNA pseudoknots

For different segments of tmRNA pseudoknots [17], the secondary structure is often captured in the structures and many of the pseudoknots are also predicted (*e.g.*, Fig. 1d). With tmRNA, different Kuhn lengths are required for each of these pseudoknots. This makes prediction of the full tmRNA sequence less successful.

The arrows in Fig. 1 indicate that the traditional secondary structure can be predicted as the stable minimum free energy in these calculations. The pseudoknot linkage stem (see Text S1) can be predicted at effectively any convenient time after the secondary structure is folded in such examples. Thus, in a 5′ to 3′ progressively folding scenario, these structures appear to be tuned to fold correctly and can easily catch the pseudoknot at the minimum free energy. There appear to be many classic pseudoknots that tend to follow this pattern of folding. Vsfold5, by utilizing this folding heuristic model, easily reveals this feature in many pseudoknots.

### Viral frame shift PK structures

The tandem frame shift pseudoknots of the tobacco mosaic virus (TMV) [7] are predicted for a minimum PK stem length 4 nt and a minimum stem length 3 bp (Fig. 1e). Using the same parameters, the turnip yellow mosaic virus (TYMV) is also predicted (Fig. 1e) [5].

The main structure of the SARS frame shift structure is also found (Kuhn length 5 nt), though the embedded stem appears to be shifted (Fig. 2a) [8], [9]. The difficulties reported in measuring and analyzing the structure of SARS may reflect a composite structure of this region in which both structures exist (possibly in equilibrium). We expect the reported structure to be the most stable. However, the clear imino-proton signal is consistent with either structure and much of the cleavage data appears to fit either structure. Perhaps developing a more detailed approach to analyzing the local structure and fine tuning of the parameterizations will favor the reported structure. An alternative structure is also shown for the case where a Kuhn length of 4 or 6 is used (Fig. 2a). This latter structure resembles the tandem pseudoknots of TMV.

Almost all the viral frame shift sequences we studied appear to have a fairly large diversity of structural morphologies. Some of these alternative structures may actually represent alternative states of the structure that are used to decide the frame shift mode. With the development of suboptimal structure prediction, a model as versatile as vsfold5 can study these energy differences more closely.

### G-box pseudoknot

For sequences of order 200 nt, the approach appears to be able to capture the correct structure for the G-box structure (predicted in its entirety Fig. 2b) [18]. An additional pseudoknot is also suggested (right hand side of the Figure). The pseudoknot for G-box can be predicted using a Kuhn length of 4 to 5 nt and invoking the effective Flory option.

### Group I intron pseudoknot

For sequences of order 400 nt, we have differing results. For the group I intron, we found the approach could predict the pseudoknot between the P3 and P6 stems successfully (Fig. 2c) [19]. However, the Flory model [20] and/or the McKenzie-Moore-Domb-Fisher model (Ref. [13]) becomes important for long compact functional RNA structures where polymer-solvent interactions begin to dominate. In Fig. 2b, the structure is fitted using *δ* = 1.4, *γ* = 1.5 and default parameters for the effective Flory model. The parameter *δ* reflects the extent or weight of the correlation in the polymer chain: a large value for *δ* indicates that the memory of the previous monomer dies off very rapidly and a small value suggests that the information persists in the structure over a much longer distance. The default parameter is *δ* = 2 (the Gaussian distribution). The parameter *γ* scales the volume occupied by a polymer in accordance with a self avoiding random walk [13]. This suggests that long sequences with extended interactions also tend to have more correlation over the entire sequence and the dimensionality may be slightly reduced. Many of these structures fold correctly with a variety of parameter values, but long sequences appear to favor smaller values for *δ*, and *γ*.

### Structures not immediately successful with the modeling heuristics

Not all sequences appear to be fully successful within this scheme.

**Figure 3:**
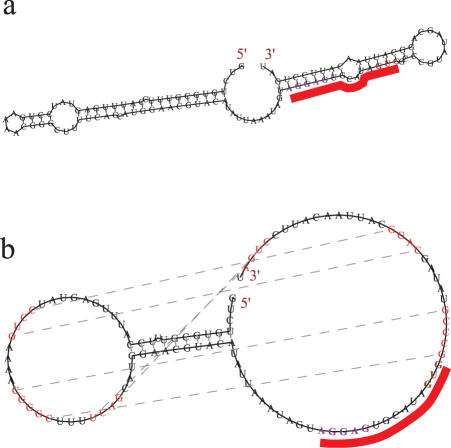
Example of a two state system. The alpha operon ribosomal binding site [21] is one example where the minimum free energy of the bare RNA is different from that of the observed structure bond to the ribosome. (a) A sequence from *E. coli* fitted with a Kuhn length of 7 nt. (b) The same sequence under constraints. The correct pseudoknot could only be obtained when the Shine-Dalgarno sequence region and the upper stem regions in the loop (Fig. 3a) were excluded.

First, the minimum free energy can only be used to predict the ground state structure and structures not altered by protein interactions. In Fig. 3, the pseudoknot associated with the alpha operon ribosome binding site (http://www.sanger.ac.uk/cgi-bin/Rfam/getacc?RF00140) is shown. For a typical Kuhn length of 7 or 8 nt, the structure takes a form shown in Fig. 3a. This occurs for many of these sequences. The reported structure is obtained when we force open the large loop structure shown in Fig. 3b [21].When forced in this way, the structure in Fig. 3b appears to be the minimum free energy. The free energy difference between the two structures is approximately 7 kcal/mol (at 37°C). The reported activation barrier for the two conformations is Δ*H* = 12 kcal/mol [21]. Including the entropy contribution from the model, this is approximately the correct order of magnitude. Therefore, this would suggest that in the fast quenching preparation that was used, obtaining a distribution of RNA structures ranging 7 kcal/mol is not unusual. The ribosome may provide a major part of the binding interaction. Since vsfold5 only predicts the minimum free energy in normal operation, for this structure, only results like Fig. 3a are expected (for Kuhn length around 7 nt or greater).

Second, we find with the use of [Mg(H_2_O)_6_]^2+^ localization [22] and the Flory model [20], we can obtain parts of *E. coli* RNaseP successfully. The actual structure has highly organized coaxial and parallel stem stacking [23]. The S-domain of RNase P can be fit almost perfectly with minimum stem length 3 bp and the [Mg(H_2_O)_6_]^2+^ option. Other global features of the complete sequence can be obtained with similar parameters as the group I intron. To obtain the whole structure, more consideration for very complex coaxial stacking arrangements and parallel stem construction will be needed to stabilize the known structure. Recent indications are that parallel alignment of stems is rather common in functional RNA [24]. The structural mapping design of vsfold5 permits development of advanced coaxial stacking and parallel stem arrangement in the form of modules and methods.

When parts of the structure are forced using constraints, we find that the FE difference between the predicted structure of RNase P and the correct structure is only 3 kcal/mol. The extensive coaxial stacking and parallel stem stacking interactions are likely to contribute at least this much to the free energy of the structure. The P1 and P3 stems are easily obtained with vsfold5, but P2 and P7 (the neck of the structure) appear quite difficult to obtain without introducing corrections that address these complex coaxial stacking and parallel stem configurations as well as permitting a variable Kuhn length in the design of the program.

### Observations

Several important observations are revealed in the modeling heuristics.

First, we see that successful predictions could be obtained by a progressive 5′ to 3′ folding strategy for quite a few RNA structures. This suggests that many important pseudoknots fold up in a hierarchical fashion like most secondary structure. The primary difference is that the pseudoknot will form somewhat concomitant with the progressive folding of secondary structure on time scales observable using NMR spectroscopy. Cooperative folding may reflect the time scale of the measurement and the sequence lengths more than the process in these examples. The approach used in vsfold5 is consistent with the hierarchical folding hypothesis [25] and adds further weight to its importance in RNA folding. It is also consistent with contact order models [26]–[28] in that longer sequence lengths take longer to fold.

Second, we obtain deeper information about the RNA itself. Here, we can learn about the flexibility of the RNA under study using the Kuhn length which is a measure of the stiffness. Nascent RNA should have different properties from large functional RNA structures such as the ribosomal RNA. Furthermore, we detect expected polymer solvent effects (Ref. [20]) when the size and complexity of the sequences increases. This is a characteristic of mature RNA involving long sequences. On the other hand, nascent RNA is less able to develop long range structure interactions and tends to form simple structure of short Kuhn length. As a result, vsfold5 provides important structural information about the RNA under study. No other prediction approach offers any information on the flexibility of the structure. It should not be expected that one button pushed answers all further questions.

Third, the entropy model is rather stable and even crude adjustments can be used to find a good structure in many cases. Similar structures produce similar free energies with the model used by vsfold [13]. Approaches of this type help us to develop generalizations about RNA. Ultimately, this offers a direction to actually design RNA like an engineer.

Further plans to develop the software and the anticipated improvements (based on currently observed information) are outlined in detail in Text S2 (Section S2.4.8).

### Time complexity

A discussion of the time complexity of the secondary structure calculations and a theoretical explanation for the contribution from pseudoknots is found in Text S2 (Section S2.3.1). Currently, the time complexity is approximately *O*(*N* ^4.7^) independent of the pseudoknot option. This is because the secondary structure methods have not been optimized. With optimization, the pseudoknot option can achieve a time complexity of approximately *O*(*N* ^4^).

### For longer sequences

Vsfold offers a stable solution for long sequences. When the domain size becomes very large, the entropy in this model discourages the formation of such domains. The model's behavior is consistent with the hierarchical folding hypothesis [25] where it is proposed that the secondary structure tends to form first followed by the tertiary structure. Its behavior is also consistent with the contact order model [26]–[28] where it is proposed that the time it takes for a structure to fold is largely dependent on the domain containing the sequence fragment with the largest number of monomers. This model is able to answer the issues raised in Ref. [26] where it was pointed out that there appears to be a correlation between prediction and the contact order model. For vsfold, this correlation is a consequence of the entropy in the model (see Ref. [13] and related references therein). Good predictions can often be obtained with a variety of parameterizations. Hence, the model tends to be stable.

For a given instance, we cannot say that this model will definitely yield a better result than any other approach. However, for the biologist who must confront the unknown structure of a new sequence, we think this tool is definitely helpful.

### Summary

We have introduced a heuristic modeling approach to solve RNA structure including pseudoknots employing structural mapping, sequential folding, and a new entropy model including globular effects that are capable of predicting a number of important pseudoknots with the minimum free energy. The model is consistent with the experimental data and thermodynamics. The unique features of this model are the mapping, its folding strategy, and its ability to explore the role of polymer swelling and globular structure formation within the context of RNA structure. The approach shows that it is possible to develop a calculation approach that accounts for long range interactions and permits the development of modules to address them. Further, if the time window is seen as a progressive process of updating the 3′ end with the new structure, the behavior is consistent with the hierarchical folding hypothesis [25] and the contact order model [26]–[28]. Very complex secondary structures can be built if the components of the structure can be explained in terms of some recognizable folding pathway.

## Materials and Methods

A web site is provided at http://www.rna.it-chiba.ac.jp/vsfold5. The program is written in C++. The executable of vsfold5 (vsfold5++) is available upon request under the following formats: Linux (Fedora Core 2, 4), Mac (OSX 3 and 4), Microsoft Visual C++(2005) and cygwin (gcc 3.4). Requests, comments, bug reports and suggestions for meeting particular needs and interests or improving the usability of the software are certainly welcome and should be addressed to the corresponding author.

The theoretical foundations of the cross linking entropy model for secondary structure are found in Ref. [13] and related references therein. The details of the algorithm, the time complexity (computation costs), memory demands, and thermodynamics for pseudoknots are explained in detail in Text S2.

Vsfold5 has the capacity to build the same level of structural complexity as existing algorithms if an appropriate folding pathway can be discerned. Here we express several highlights of the approach.

### General description

The model assumes that the secondary structure of RNA forms in a 5′ to 3′ direction as the RNA is transcribed, where 5′ and 3′ refers to the beginning and end of the sequence respectively and is by convention drawn from left to right. Two buffers are built up: one that contains the current structure and tests for new secondary structure at the current sequence position and another that tests for pseudoknots on that same structure and sequence. The buffers compete with each other and the minimum free energy is selected from the best result: whether secondary structure or pseudoknot. As a result, the structure that is predicted is the minimum free energy for a given sequence fragment with a given persistence length, temperature and solvent condition.

In the mapping approach, there are two types of pseudoknots (PK): core PKs and extended PKs. From the perspective of folding, the major difference lies in whether the PK is formed in advance of potential secondary structure (and is more stable than the alternative secondary structure over the same interval) or is the result of fusing existing secondary structure together (extended PKs). Core PKs can also form as a result of sweeping up leftover free strand after the secondary structure has formed.

### Core pseudoknots

**Figure 4:**
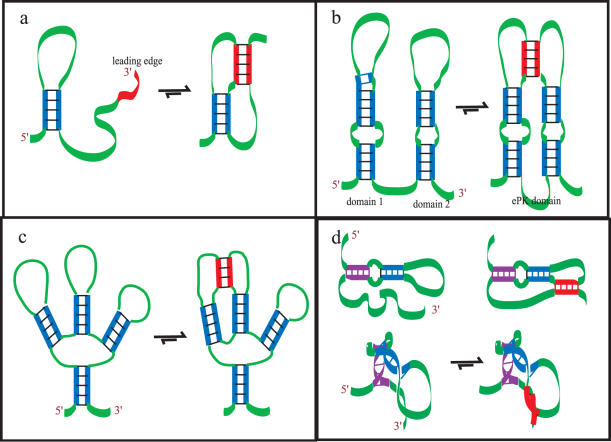
Some basic concepts of pseudoknot structure and folding that are considered in the vsfold5 algorithm. (a) Basic concept of folding for a core pseudoknot. The stable secondary structure is formed first, followed by addition of stem into the loop. The red region corresponds to the point where the linkage stem forms. (b) Basic concept of folding for an extended pseudoknot. An extended pseudoknot involves the fusing of two independent domains via a small segment of secondary structure. As in (a), this stable secondary structure is formed first, followed by joining the two independent domains into a single domain. (c) Basic concept of an embedded pseudoknot. Here, the secondary structure naturally permits a multibranch loop to form, and the extended pseudoknot that links two branches of the secondary structure is shown here in thermodynamic equilibrium with the standard secondary structure. (d) Basic concept of pleating. The secondary structure (shown above in d), appears to require a very long free strand region (green) to insure that the red segment of secondary structure is formed. However, when this is viewed more three dimensionally, the internal loop permits this structure to fold back on itself and requires a much shorter segment length.

Core pseudoknots consist of a root stem and a linkage stem that often forms at the 3′ end of the structure (the “leading edge” in Fig. 4a). In its most basic form, it is also referred to as a H-type PK or an ABAB PK [29], [30]. However, the core PK can be embellished with complex motifs of RNA structure to any extent required as long as these structures are thermodynamically stable (Text S2).

With the leading edge approach, the 5′ to 3′ folding first builds the minimum free energy (mFE) structure for the current 3′ position (from position 1 to *j*) and parses it with the leading edge sequence of a predefined length; typically about 7 to 10 nt and shorter than a simple stem-loop (approximately 2 or 3 times the Kuhn length). All of the existing structure is subject to editing around the leading edge. As the secondary structure calculations catch up to the calculated leading edge points, the leading edge has a chance to choose between recently formed secondary structure and a pseudoknot over the same length of sequence.

Alternatively, after a stable interval of structure has developed, the existing structure can then collect up the surrounding left over free strand and form a pseudoknot.

### Extended pseudoknots

An extended PK involves the joining two independent domains of secondary structure that have already formed by a small linkage stem (Fig. 4b). These have also been referred to as ABACBC PKs, but like the core PKs, extended PKs can be embellished to any extent required (Text S2). A domain of RNA structure consists of a RNA sequence fragment that forms a stable isolated secondary structure independent of the remaining sequence on the 5′ and 3′ sides of the domain. In this respect, the fragment can be snipped out and folded without major changes happening to the structure. An example of such a domain is shown in Fig. 4b where domain 1 and domain 2 could be snipped out of the sequence and each structure would persist unaffected. When a pseudoknot joins these independent domains, the entire complex becomes a single domain (Fig. 4b). The approach for extended PKs assumes that evolution has selected domains that are stable when formed during the folding process and do not change significantly even with the addition of pseudoknot interactions. This is shown schematically in Fig. 4b and is consistent with the hierarchical folding hypothesis [25]. A pseudoknot becomes part of a subdomain when it is incorporated into other secondary structure (Fig. 4c).

### Structural Considerations

Unlike secondary structure, where there is a greater amount of space separating secondary structures, the increased proximity of pseudoknot structural features require more attention to existing structure in making predictions. Only simple types of structure features are currently analyzed.

The first is pleating. When RNA can chose between a single straight stem and a group of stems that fold back on themselves, there is a good chance that the latter will be selected because of the increased interaction between neighbouring (parallel) stems. For example, structures such as the *Tetrahymena thermophila* group I intron show P5 folded back onto P4 [19]. This folding can bring an otherwise distant free strand into proximity with a pseudoknot forming structure (Fig. 4d).

Other significant interactions are configuration and orientation strain. This happens when parts of the structure must be stretched or twisted in order to accommodate the linkage stem. To accommodate such structures, it is important to minimize this strain. These features are discussed in further detail in Text S2.

## Supporting Information

Text S1 A brief introduction to RNA secondary structure definitions for the beginner. Provided for readers who wish to understand the basic concepts of RNA secondary structure including pseudoknots and knots.
(0.08 MB PDF)

Text S2 Methods, a description of the methods used in vsfold, the time, memory and structural complexity, and the thermodynamics. Provided for readers interested in knowing how the mapping algorithm in vsfold5 works for handling secondary structure and pseudoknots. The calculation of pseudoknot stability and 3 dimensional considerations, the time complexity and the pseudoknot complexity are described or explained here.
(0.68 MB PDF)
