# Supplementary material for: Prediction of RNA Pseudoknots Using Heuristic Modeling with Mapping and Sequential Folding
Source: PLoS One. 2007 Sep 19;2(9):e905. doi: 10.1371/journal.pone.0000905 (PMC1975678; doi:10.1371/journal.pone.0000905)
Supplement: Text S1 — A brief introduction to RNA secondary structure definitions for the beginner. Provided for readers who wish to understand the basic concepts of RNA secondary structure including pseudoknots and knots. (0.08 MB PDF) [file pone.0000905.s001.pdf]

Supplement S1: RNA secondary structure

RNA structure is often expressed schematically by its base pairing: the Watson-Crick (WC) base pairs A (Adenine) with U (Uracil), and G (Guanine) with C (Cytosine) and also the non-Watson-Crick (non-WC) base pair G with U. RNA sequences are typically written from left to right. The beginning of the sequence is usually on the left hand side and is called the 5’ end, and the opposite end is called the 3’ end.

A number of simple structural motifs are generated by the way that the RNA molecule forms these base pairs. In secondary structure of RNA, the typical structural motifs are usually classified as stem, internal loop (or interior loop), and multibranch loop. This standard neglects a large group of possible structures called pseudoknots (PK).

Stem:

When more than one base pair appears in the form of a group of contiguous base pairs, the resulting structure motif is described as a stem (Fig. S1a). For RNA, this stem motif appears as a flat object. However, the actual structure in three dimensions (3D) has a twist that makes a 360° rotation roughly every 10 bps (for A-RNA: the most commonly found structure for RNA).

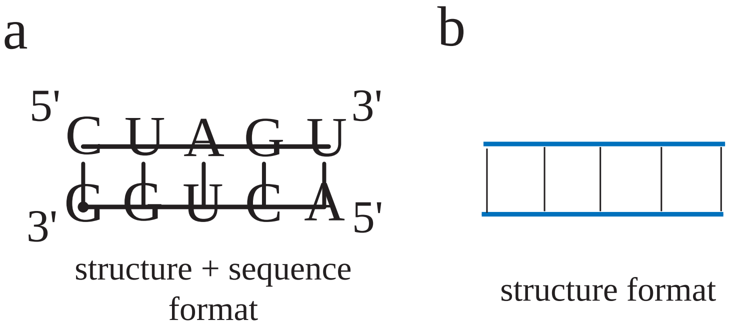

Supplement Figure S1. An example of a stem motif represented as secondary structure. (a) A stem including both the secondary structure and the sequence labels. (b) A stem that only includes the base pairing information.

Loops:

The other simple structural motifs, based on the types of loops, can also be found in RNA structures.

Hairpin loop (H-loop):

The simplest such motif is the H-loop (Fig. S2). The H-loop consists of two complimentary sequences joined by some non-pairing bases in a loop. A simple example would be the sequence

AAAAACCCCUUUUU (Fig. S2a). The Figure also contains three additional representations. In Figure S2b, only the base pairing information of the structure diagram is shown (in a similar way as Fig. S1b). Figure S2c is a bracket graph developed by Hofacker *et al.* [1], where the bracket is matched by an equal and opposite bracket on the 3' side. Figure S2d is a highly simplified diagram similar to Fig. S2b, but lacking specific details about the exact size of the loop region.

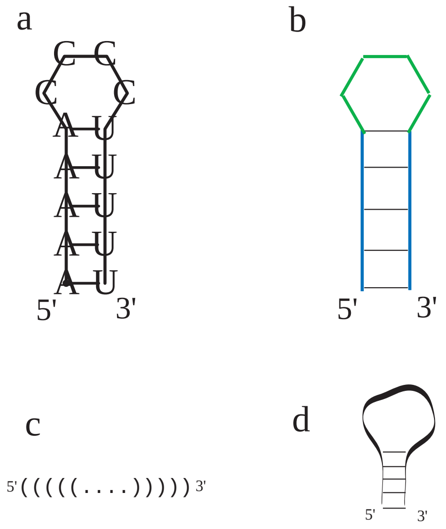

Supplement Figure S2. An example of a hairpin loop (H-loop). (a) Secondary structure with base index included. (b) Secondary structure with only the base pairing and base position included. (c) A simplified representation of this secondary structure using bracket notation. (d) A secondary structure that only specifies the stem base pairs without specifying the exact size of the loop regions.

Internal loop (I-loop):

Another common structure is the internal loop (I-loop), Fig. S3a-h. An internal loop appears between two stems and has n_1 unpaired bases on the 5' side and n_2 unpaired bases on the 3' side; where $n_1 = 0,1,2\ldots$ nucleotides (nt) and likewise for n_2. Fig. S3a shows a symmetric internal loop ($n_1 = n_2$) where $n_1 = n_2 = 1$ nt: *i.e.*, the number of bases in on each side of the loop are equal to one. This I-loop motif also includes bulges which have the property that $n_1 > 0$ and $n_2 = 0$ or $n_1 = 0$ and $n_2 > 0$. Large structures of RNA typically have many loops, bulges, and internal loops. There are many examples of symmetric I-loops found in RNA structure databases. Some asymmetric I-loops ($n_1 \neq n_2$) can also be found.

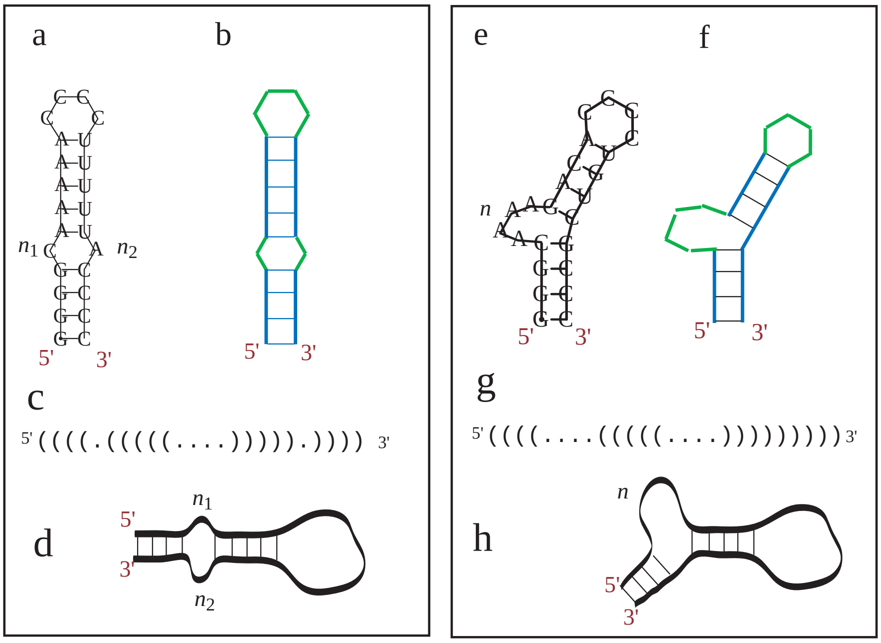

Supplement Figure S3. Examples of secondary structure for internal loops and bulges (I-loops). (a) Secondary structure of internal loops with base index included. (b) Secondary structure with only the base pairing and base position included. (c) A simplified representation of this secondary structure using bracket notation. (d) A secondary structure that only specifies the stem locations and is not specific about the loop regions. (e-h) The same definitions apply for the example of a bulge.

Multibranch loop (M-loop):

A third common secondary structure motif is known as a multibranch loop (M-loop or MBL). These are more complex structures that consist of several of these previously described stem-loop type structures. These structures are also quite common like the other secondary structure motifs. An example of a multibranch loop is shown in Fig. S4.

The stem, H-loop, I-loop and M-loop are the four fundamental motifs that make up secondary structure. The fundamental feature of RNA secondary structure is that base indices i and j ($i < j$) are allowed to base pair with each other if they satisfy the following properties with all other base pairs (i' and j', with $i' < j'$)

(1) if i and j are contained within i' and j', then $i' < i < j < j'$

(2) if i and j are not contained within i' and j', then either i and j are less than i', or i and j are greater than j',

(3) if neither case is true, then $i = i'$ and $j = j'$.

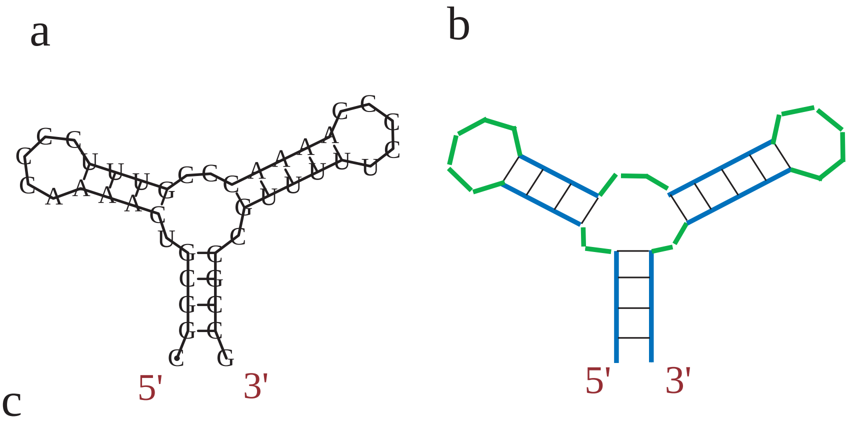

c

5' $.((((.((((.....))))..(((((.....))))).)))).$ 3'

Supplement Figure S4. Secondary structure of multibranch loops (M-loop or MBL). (a) Secondary structure of M-loop with base index included. (b) Secondary structure with only the base pairing and base position included. (c) A simplified representation of this secondary structure using bracket notation.

Pseudoknot (PK) and knots:

Whereas many RNA structures are known to satisfy these rules (for example, tRNA), this is not always the case. The most common deviations from these base pairing rules is a class of structures called pseudoknots (PKs). A pseudoknot permits violation of the above three pairing relationship rules.

(4) For pseudoknots: parts of the structure still satisfy cases 1 through 3. However, in addition, in at least one part of a region between k and l such that $k \le i, j, i', j' \le l$, there exists some base pairs that satisfy either $i' < i < j' < j$ or $i < i' < j < j'$.

Hence, many more possibilities can be generated once we start allowing pseudoknots.

Pseudoknots differ from real knots in the sense that the strand does not pass completely through the loop but only becomes potentially entangled with it. Anyone who has tried to untangle a pair of earphones or tried to untangle a recently, neatly wound up cord can realize that knots naturally occur on long flexible cords. Indeed, great effort seems to be required to avoid tangling a heap of cords.

Figure S5a shows a common pseudoknot known as an H-type pseudoknot. This is also known as an ABAB pseudoknot. The structure is notated below with the standard parenthesis notation for the basic secondary structure (here shown in blue) and square brackets for the pseudoknot linkage (here shown in red). Green indicates the regions of free strand that are not forming base pairs (bps).

The color distinction of the stems in this example is not important because both stems are the same length and both stems are less than 10 bps.

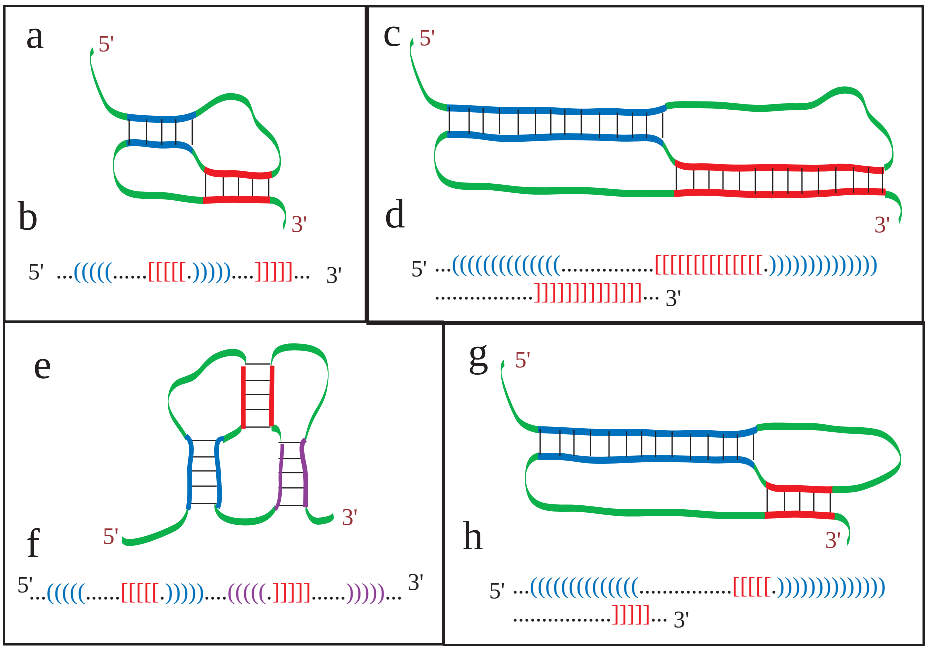

Supplement Figure S5. Examples of pseudoknots and knots. (a) An H-type pseudoknot where the linkage stem is denoted in red, standard secondary structure in blue and free strand (regions of no base pairing) in green. (b) The same structure in (a) denoted in bracket notation. (c) An example of a knot where the stem length of both the secondary structure and the linage stem are longer than 9 base pairs. (d) The same structure in bracket notation. (e) An extended pseudoknot, sometimes referred to as a kissing-loop and also known as an ABACBC pseudoknot. (f) The structure shown in bracket notation. (g) A pseudoknot. The difference between (c) and (g) is that the linkage stem is shorter than 9 contiguous bps. (h) The same structure in bracket notation.

Figure S5c shows a knot and the corresponding bracket structure is shown below in Fig. S5d. Both stems in Fig. S5c contain 14 base pairs (bp). Since the helical axis makes a rotation of 360° every 10 bps, this means that the structure in Fig. S5c is tangled in a knot. There is no reason why such a structure cannot form. Indeed, knots are known to form in some rare proteins [2,3]. However, it is not a pseudoknot and the current approach is not designed to estimate its existence or the likelihood of its formation. One important feature of a pseudoknot is, therefore, that the linkage stem (here shown in red) must be shorter than 10 contiguous bps.

Figure S5e shows two stem-hairpins-loops (blue and purple) that are joined by a linkage stem (red). The stems are all short as in Fig. S5a. This is a pseudoknot, sometimes referred to as a kissing

loop. It is also known as an ABACBC pseudoknot. It is also observed in a number of places, although less frequently than H-type pseudoknots. The corresponding bracket notation for this structure is shown below (Fig. S5f).

Figure S5g shows a structure intermediate between Figs. S5a and c. This structure is also an H-type pseudoknot. Here we see a necessary condition for defining a segment as a linkage stem and the most important distinction between a knot and a pseudoknot: a linkage stem cannot contain more than 9 contiguous bps. When an internal loop breaks the continuity, this rule may not necessarily apply. Therefore, longer overall structure could form, but only if the stem is not contiguous. Moreover, this would have to be considered on a case by case basis.

As a final curiosity, in Fig. S6, a secondary structure (left hand side) and its corresponding knot (right hand side) are shown in equilibrium. The knot renders a peculiar 2D illusion perhaps reminiscent of artist M.C. Escher's "Belvedere" (1958). The knotted structure could conceivably exist in chemical equilibrium with a standard secondary structure, though electrostatic effects may render it less favorable thermodynamically. It could result from a simple misfolding.

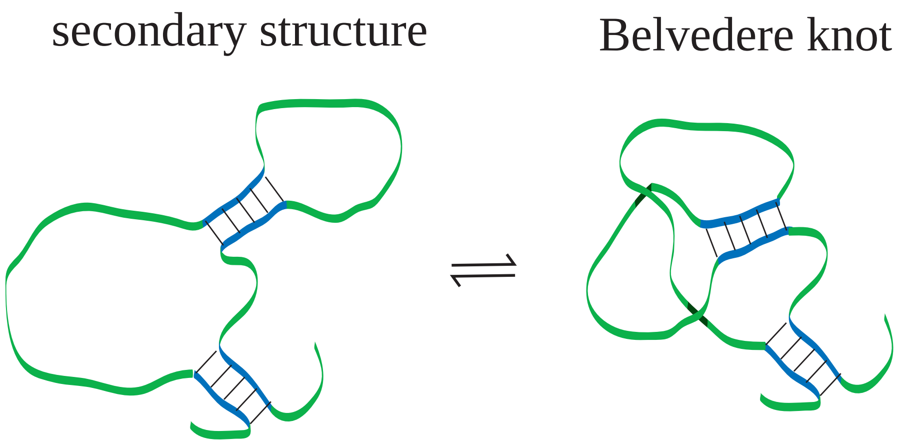

Supplement Figure S6. A special type of knot (right hand side) that becomes entangled due to the way the structure folds up. The two dimensional nature of the RNA schematics tends to hide curious possibilities as this. The knot is seen in equilibrium between the unknotted structure (left hand side) and the "Belvedere knot" (right hand side).

Functional RNA structures that contain knots of the form shown in Figs. S5c or S6 are currently unknown or have not been reported. However, pseudoknots are often observed in functional RNA structures, particularly H-type pseudoknots (Fig. S5a). Single strand RNA sequences such as messenger RNA with introns can have sequences with lengths that number in the tens of thousands of nucleotides. With such a propensity for a few simple cords to become tangled, and, since the cell

can have many thousands of protein and RNA strands present within the cellular environment, this suggests that there is a fair amount of effort made within the cell to prevent or get rid of knots [2].

In this work, we are concerned with the prediction of pseudoknots. These structures have the property that they can be evaluated as structures resulting from reversible folding.

References

1. Hofacker IL, Fontana W, Stadler PF, Bonhoeffer S, Tacker M, *et al.* (1994) Fast folding and comparison of RNA secondary structures. Monatshefte f Chemie 125: 167-188.
2. Lua RC, Grosberg Y (2006) Statistics of knots, geometry of conformations, and evolution of proteins. PLoS Comp Biol 2: e45.
3. Virnau P, Mirny LA, and Kardar M (2006) Intricate knots in proteins: function and evolution. PLoS Comp Biol 2: e122.
