# Supplementary material for: Prediction of RNA Pseudoknots Using Heuristic Modeling with Mapping and Sequential Folding
Source: PLoS One. 2007 Sep 19;2(9):e905. doi: 10.1371/journal.pone.0000905 (PMC1975678; doi:10.1371/journal.pone.0000905)
Supplement: Text S2 — Methods, a description of the methods used in vsfold, the time, memory and structural complexity, and the thermodynamics. Provided for readers interested in knowing how the mapping algorithm in vsfold5 works for handling secondary structure and pseudoknots. The calculation of pseudoknot stability and 3 dimensional considerations, the time complexity and the pseudoknot complexity are described or explained here. (0.68 MB PDF) [file pone.0000905.s002.pdf]

Supplement S2: Methods: the vsfold5 algorithm

Here we present the basic concepts behind the vsfold5 approach. In Section S2.1, we briefly review what algorithms have been (or are being) used to address the pseudoknot problem (for reference). Section S2.2 explains how the maps define the secondary structure and pseudoknot structure. In Section S2.3, the time, memory and structural complexity are described. Finally, Section S2.4 provides a general explanation of the concepts that are used to estimate the free energy when pseudoknots are considered.

Vsfold uses a dynamic programming algorithm (DPA) to find the minimum free energy (mFE) using the cross linking entropy (CLE) model [1]. For solving pseudoknot (PK) problems, a method of handles, exit tags and maps has been developed. In addition, many tools are built around these handles and mapping methods. These tools permit a methodology that considers the context of the structure. In total, these techniques permit back editing of structural data and free energy (FE) data.

The details of the entropy and FE contributions to PK formation are expected to advance well beyond the rather simple models developed here. The current discussion on the model is meant to show that this modeling approach can be used to determine any form of structural information that exists to at least the level of resolution that are built into the model itself.

S2.1. Existing pseudoknot algorithms: a brief history

Techniques for solving pseudoknots fall into roughly six widely overlapping categories.

Loop matching strategies by and large are the oldest and currently are still utilized with other techniques. Early methods employed free energy to weight the loops [2,3] and Monte Carlo techniques [2] to find the best pseudoknot. A recent developed approach (PLMM_DPSS) employs free energy weight of loop fragments and special features of pseudoknots to discriminate likely structures and reports computation times (known as time complexity) of order $CN^2 \log N$ and N^2 in memory, where N is the number of nucleotides in the sequence and C is a constant [4]. Similarly, HotKnots is a heuristic approach that builds up candidate structures by adding fragments of secondary structure one at a time (from secondary structure calculations) and allows multiple partially formed structures [5].

Folding also has a long history, particularly the genetic algorithm [6]. More recently, Cao *et al.* have developed a realistic lattice model of the RNA sequence backbone [7]. Likewise, KineFold is an elaborate local folding model that handles sequential 5' to 3' folding [8,9]. Notable in these latter two methods is that realistic considerations about the three dimensional (3D) structure of a pseudoknot and polymer dynamics are seriously considered in the models. However, convergence for all these folding algorithms is typically slow and except for Cao *et al.* (which is N^6), not

necessarily guaranteed.

Other techniques consist of alignment of sequence or structure. One of the earlier systematic approaches employed maximum weight matching (MWM) for sequence alignment including pseudoknots [10]. More recently, dynamic MWM [11] has also been proposed. These can run rather fast, but suffer some loss of accuracy. A hybrid method of loop matching with thermodynamics and sequence alignment known as iterative loop matching (ILM) is a significant extension of the original MWM approach [12]. PSTAG is a structure alignment technique using pair stochastic tree adjoining grammars [13]; an approach that has its origins in studies in language processing [14]. A language processing strategy is potentially powerful [14,15]; however, they are rather costly in time complexity (N^5) and memory (N^4) [13]. A simplified approach that applies only the parsing part of this procedure was also developed with some success [16]. Of these alignment strategies, ILM claims the smallest time complexity (N^4) and memory (N^3).

Recently, a hybrid method has been developed that employs other methods to gain a consensus for H-type pseudoknots [17].

The last two groups involve exact thermodynamic models that follow in the tradition of the secondary structure calculations. Of these, the first group finds only the mFE with no suboptimal structures. The most precise model, PKNOTS, that guarantees the minimum free energy is computationally costly in time (N^6) and memory (N^4) [18]. Recently, a simplified approach has been developed that sacrifices some detail by applying canonical rules but only requires a time of N^4 and memory N^2 [19]. Knowledge of the structural features of typical pseudoknots is used in this approach to help in the search and reduce the search space [19]. The final strategy follows in the traditions of partition functions for secondary structure [20] and therefore provides the possibility of suboptimal structure prediction [21].This approach sacrifices some detail and has a costly time complexity (N^6).

Of these approaches, in general, thermodynamics has been the poorest performer both in prediction [22] and generally at finding the correct mFE of any structure [19].

Vsfold4 is a thermodynamic method that finds the minimum free energy [1], uses the sequential 5' to 3' folding direction of a biological context, and uses the cross-linking entropy model (a theoretical model [1]) to find the structure. It has scored close to 80% success on secondary structure predictions of a complete genome of tRNA sequences, which approaches the typical success of sequence alignment approaches. However, vsfold4 can only calculate secondary structure, which restricts its utility. The current version, vsfold5, has made the important leap to predicting pseudoknots, and, at the same time, does not add significantly to the overall time complexity of the calculation.

S2.2. Vsfold5 Mapping methods

Vsfold5 uses pointers to map the secondary structure, and handles and a lookup table of exit tags to link different segments of secondary structure together to form different parts of the pseudoknot. There are other formal mapping methods [23], but for the problems that are addressed here, the approach explained in this work appears to be more applicable.

Here we define the indices i and j represent residues (base i and base j) in the RNA sequence with $i < j$ and the indexing runs successively from the 5' end to the 3' end of the sequence. A base pair (bp) that is formed between base i and base j is indicated by the ordered pair (i, j).

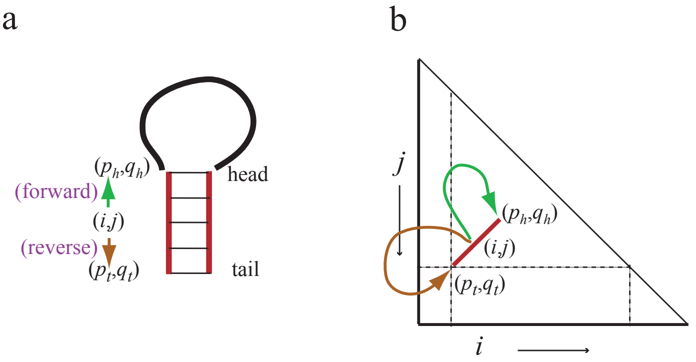

Supplement Figure S7. Characteristics of a pointer located in a stem, or when $(i, j) \Leftrightarrow (p_h, q_h)$ at a hairpin loop. A basic stem consists of a tail located at (p_t, q_t) and a head located at (p_h, q_h). For the pointer index at (i, j), a forward pointer (green arrow) points to (p_h, q_h) and a reverse pointer (brown arrow) points in the opposite direction at (p_t, q_t). (a) A basic stem and hairpin attached to the stem at (p_h, q_h). (b) The triangle diagram representing this hairpin loop with the pointer index (i, j) and the forward (green arrow) and reverse (brown arrow) pointers.

S2.2.1. Secondary structure pointers

In this approach, the pointer maps the next major position of the secondary structure and any previous position that is present in the structure. The pointer consists of the current position (i, j), a tag classifying the type of secondary structure located at (i, j), a forward link (labeled "forward" in Fig. S7a) and a reverse link (labeled "reverse" in Fig. S7a). The essential secondary structure tags consist of the following: 'S' (part of a stem), 'I' (internal loop or a bulge), 'H' (hairpin loop), and 'M' (multibranch loop). Stems are further divided into main stems (tag 'S') and short stem fragments ('C', usually 2 base pairs). The reason for this minor distinction is that these short fragments rarely

appear alone outside the context of a neighboring stem of longer length.

For example, Fig. S7a shows a stem with a pointer located at a base pair (i, j). The tag at (i, j) is 'S', the forward pointer (green arrow labeled "forward") aims at the head of the stem (p_h, q_h) and the reverse pointer (brown arrow labeled "reverse") aims at the tail of the stem (p_t, q_t). When the pointer is located at (p_h, q_h) in the stem (same Figure), it points to the next secondary structure and back to the tail of itself at (p_t, q_t). In Figure S7, the next secondary structure is a hairpin (tag 'H') and the pointer points forward to itself at (p_h, q_h). Figure S7b shows a triangle diagram representation of the secondary structure and the respective coordinates for a stem terminated by a loop (known as a "stem-loop").

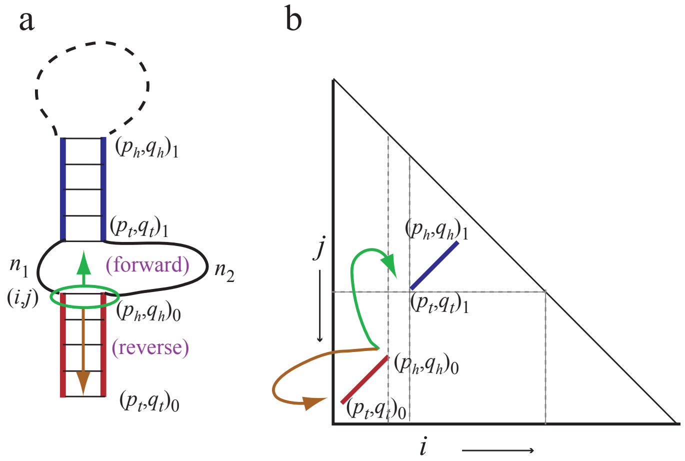

Supplement Figure S8. Characteristics of a pointer located at an internal loop. Here, the pointer (i, j) is located at $(p_h, q_h)_0$. The forward pointer (green arrow) points to $(p_t, q_t)_1$ and a reverse pointer (brown arrow) points in the opposite direction at the tail of the current stem $(p_t, q_t)_0$. (a) A basic internal loop attached to a stem at $(p_h, q_h)_0$. (b) The triangle diagram representing this internal loop with the pointer index (i, j) and the forward (green arrow) and reverse pointers (brown arrow).

In Figure S8a, (i, j) is located at the head of stem $(p_h, q_h)_0$, indicated also by the green oval. The secondary structure is an internal loop (tag 'I') and the forward pointer aims at the tail of the next stem located at $(p_t, q_t)_1$; this is also shown in Fig. S8b as a triangle diagram. Like the hairpin loop, the reverse pointer indicates the tail at $(p_t, q_t)_0$. (The subscript index is used for notation purposes here. The pointers at each position (i, j) are sufficient to fully index all necessary information.) In Figure S9a, the pointer (i, j) is at a multibranch loop (tag 'M') at $(p_h, q_h)_{00}$ (green oval). Here, a single pointer is insufficient to indicate the branching of the stem and an array of pointers is applied at $(p_h, q_h)_{00}$ to map out the branches at $(p_t, q_t)_{11}$ and $(p_t, q_t)_{12}$:

forward 1 and forward 2 respectively. The reverse pointer aims at $(p_t, q_t)_{00}$ whose tag is 'S'. The pointers are also shown in Fig. S9b with a labels "(f1)" and "(f2)" indicating the forward pointers (green arrows), and "(r)" indicating the reverse pointer (brown arrow).

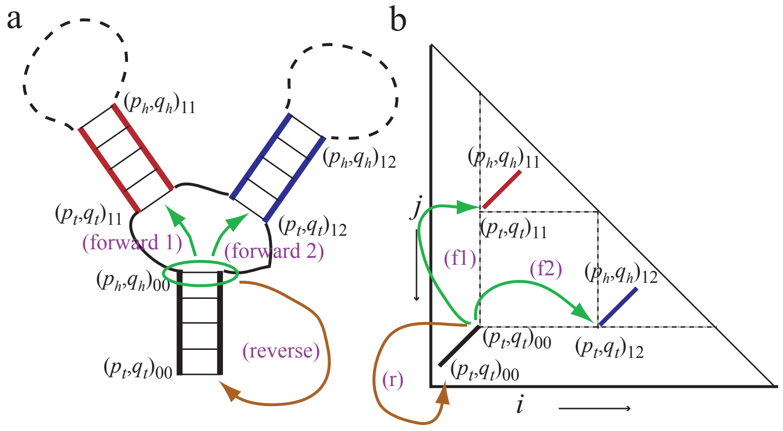

Supplement Figure S9: Characteristics of a pointer located at an multibranch loop. Here, the pointer (i, j) is located at $(p_h, q_h)_{00}$. The forward pointers (green arrows) points to $(p_t, q_t)_{11}$ (forward 1) and $(p_t, q_t)_{12}$ (forward 2) and a reverse pointer (brown arrow) points in the opposite direction at the tail of the current stem $(p_t, q_t)_{00}$. (a) A basic multibranch loop attached to a stem at $(p_h, q_h)_{00}$. (b) The triangle diagram representing this multibranch loop with the pointer index (i, j) and forward "f1" and "f2" pointers (green arrows) and a reverse pointer "r" (brown arrow).

Finally, when the pointer is located at (p_t, q_t), it points forward to (p_h, q_h) and points in reverse to the nearest secondary structure that connects it, or to itself if there is no secondary structure connecting it.

In short, the forward pointers map the structure at (i, j) to any connecting indices (i', j') that satisfy either $i < i' < j' < j$ or $i = i' < j = j'$. Any information within the boundaries can be discerned from these mapping pointers. Likewise, when we want to discover information about other parts of the structure $\{i', j' < (i, j)$ or $(i, j) < i', j'$ or $i' < (i, j) < j'\}$, the reverse pointers help find this information about the rest of the domain and, when combined with the forward maps, can discern information about every part of the existing structure.

This information is regularly updated with each construction of the map or section of the map.

A summary of the secondary structure pointers is shown in Tab. S1. For RNA secondary structure, this information is sufficient to map a complete structure and assess the relationship of a given secondary structure at (i, j) to other secondary structure in other regions of the map. The

modeling provides more immediate structure information than traditional methods. Currently, most of the secondary structural considerations in vsfold5 are limited to the local region in the vicinity of (i, j) where the main focus is on the persistence length and stem formation. However, with pseudoknots (next section), global search methods were required because the configuration information about other parts of the structure aid in deciding the stability of any potential structure. Modules are easily added to expand this functionality further.

Legal tag(s) (`ss_name`)	forward pointer (`ss_link`)	reverse pointer (`ff_link`)	Multiloop branches (`ss_iMBL`)	Figure examples
'S' (or 'C')	(p_h, q_h)	(p_t, q_t)		S7
'H'	(p_h, q_h)	(p_t, q_t)		S7
'I'	$(p_t, q_t)_1$	$(p_t, q_t)_0$		S8
'M'	$(p_h, q_h)_{00}$	$(p_t, q_t)_{00}$	$(p_t, q_t)_{11}$, $(p_t, q_t)_{12} \cdots$	S9

Supplement Table S1. List of pointers used in the secondary structure maps: (`ss_name`) a tag indicating the secondary structure, (`ss_link`) the forward link, (`ff_link`) the reverse link and, (`ss_iMBL`) the array of pointers linking the pointer to the branches of the MBL. (Example Figures): The corresponding Figures used for these examples. The tags represent the following secondary structure: 'H' hairpin loop, 'I' internal loop, 'M' multibranch loop (MBL), 'S' stem and 'C' connect stem.

S2.2.2. Pseudoknot handles

The pseudoknot (PK) adds considerable complexity to the simple pointer type mapping used in secondary structure calculations. To address this, handles are used to map out the pseudoknot superstructure, while the intervening secondary structure is handled in the same way as before. With the exception of true knots, if some folding pathway can be described by decomposing the structure into a set of sub-structural modules, all typical types of pseudoknot structures can be described by this approach.

First we distinguish between two types of pseudoknots: core PKs and extended PKs.

S2.2.2.1. Core pseudoknots (cPK):

A core PK consists of a domain (or a group of domains) of secondary structure that are joined by a linkage strand. Its primitive structure resembles an H-type PK [7] or in other terminology, an ABAB PK [24]. This is expressed by the outlined structure (Fig. S10a, the outline colored blue, orange and red and extending from the labels i_R and j_R). The gray structures indicate additional

secondary structure that could embellish this core PK and are mapped onto the core PK structure by the handles. A triangle diagram of the same structure is shown in Fig. S10b.

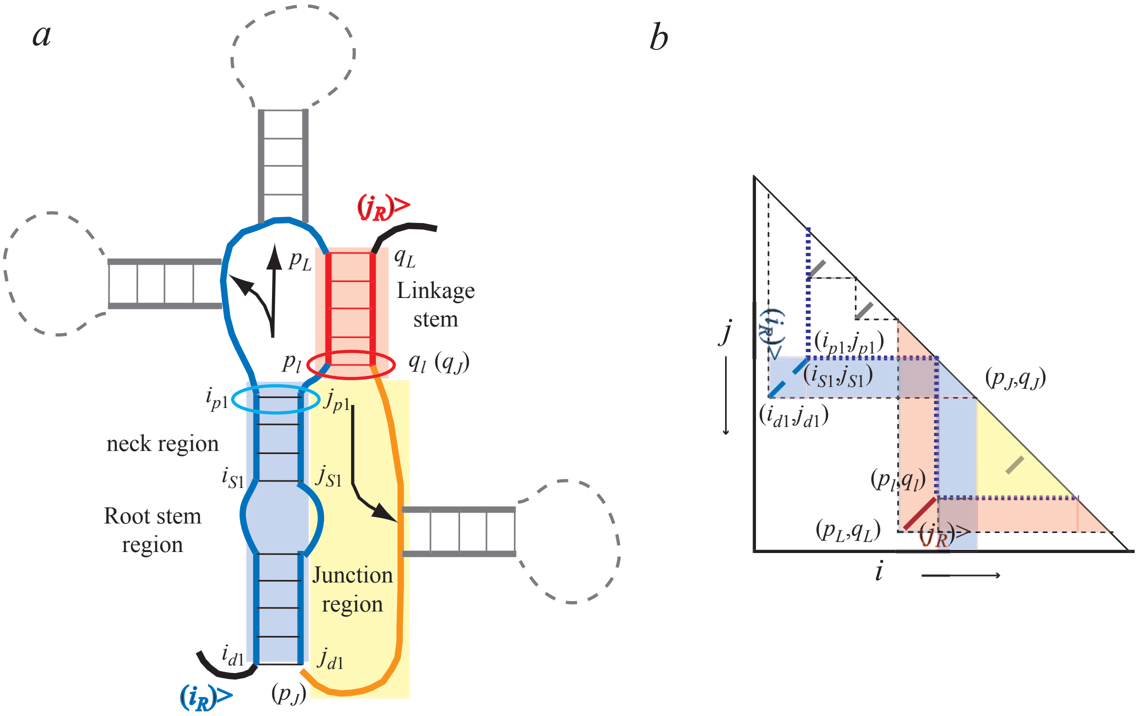

Supplement Figure S10: Example of a core pseudoknot: (a) the pseudoknot structure and labels, (b) the corresponding structure on the triangle diagram. The gray stems extending from the main structure indicate some of the multitude of possible embellishments that could be added to the basic structure.

The core PK handle contains an input index (i_R) and output index (j_R) forming a complete independent module. To (i_R, j_R), other secondary structure (ss) pointers or PK handles can be mapped onto this handle. This permits PKs that are embedded (or nested) into other ss and PKs.

The handles express the main coordinate for the root stem (i_{d1}, j_{d1}) (Fig. S10a, region with blue background), the junction region (p_J, q_J) (region with yellow background) and the linkage stem (p_L, q_L) (region with red background). To this, a neck region that contains a stem with tail at (i_{S1}, j_{S1}) and head at (i_{p1}, j_{p1}) is chosen on the root domain closest to the linkage stem to map out additional secondary structure and the local structure surrounding the point of attachment of the linkage stem. When (i_{S1}, j_{S1}) is equal to (i_{d1}, j_{d1}), then only a single simple stem is present at the root stem's domain. The junction region (p_J, q_J) builds similar maps as (i_{p1}, j_{p1}) around the junction region. The linkage stem consists of a map of linkages permitting the linkage to account

for small bulges and internal loops in the linkage caused by non-WC pairing and mismatches.

The root domain can consist of a complex domain of secondary structure, a simple stem, or another pseudoknot. The junction region and the neck of the root stem can also associate complex ss, PKs, or a simple loop. Hence, considerable complexity can be systematically mapped onto the PK.

In short, the handles map out global configuration in a similar way as the pointers map out local secondary structure, but they handle considerably more complex and detailed information than do the simpler ss pointers. Table S2 shows a description of the handles used for cPKs, where the handles used by cPKs are indicated in the upper section of the Table.

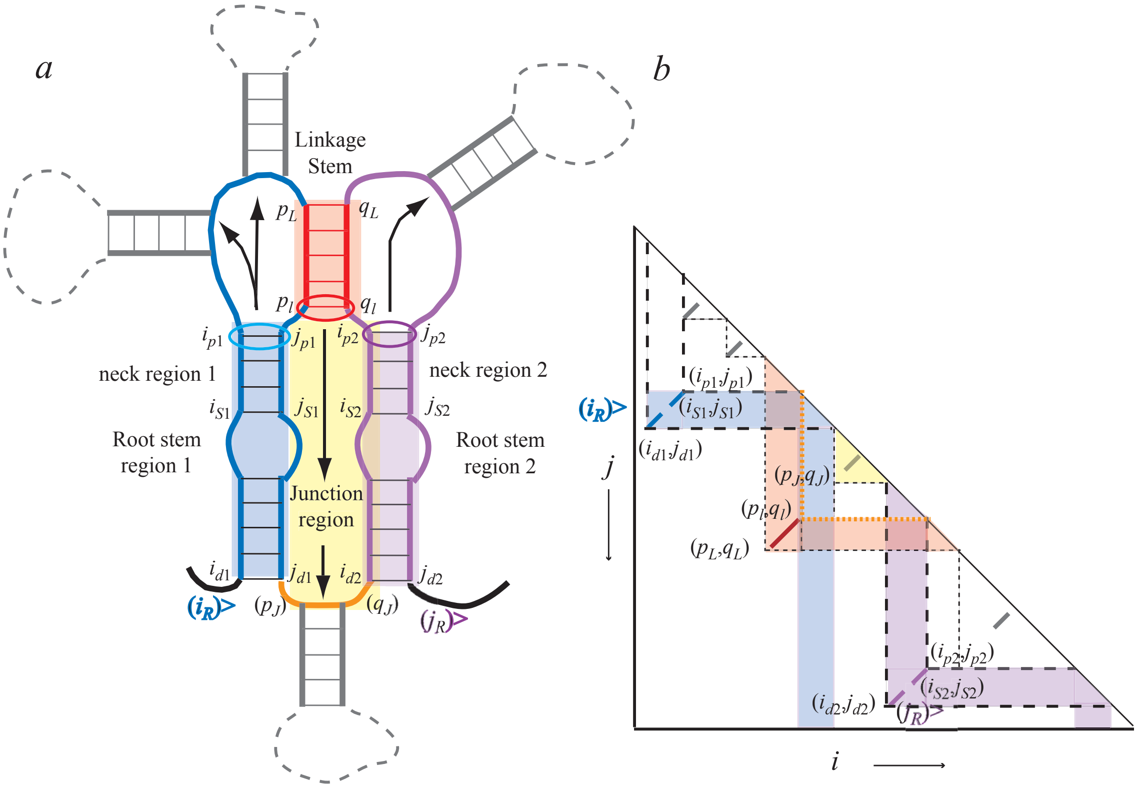

Supplement Figure S11: Example of an extended pseudoknot: (a) the pseudoknot structure and labels, (b) the corresponding structure on the triangle diagram. As in Fig. S10, the gray regions represent examples of the multitude of possible embellishing structures.

S2.2.2.2. Extended pseudoknots (ePK):

Some types of PKs cannot be mapped using the cPK architecture. These types of structures have the characteristic that they join two independently stable domains of structure (often secondary structure) together by a small linkage stem (Fig. S11a). These are called extended pseudoknots (ePK) because they extend over at least two independently stable domains of complex RNA structure. The

essential structure of an ePK is shown in Fig. S11a along with the corresponding half triangle diagram (Fig. S11b). The primitive form of an extended PK resembles the ABACBC type structures [24]. The pointers used for an ePK are summarized in Tab. S2.

Descriptor handle	Legal pointers (`pk_link`)	Legal tags (`pk_name`)	Free energy label (`fPK`)
`ijR`	(i_R, j_R)	'R' or 'K'	dGR
`pqL`	(p_L, q_L)	'L'	dGL
`Pql`	(p_l, q_l)	'H'(='J'), 'I', 'M'	dGwt
`pqJ`	(p_J, q_J)	'H'(='J'), 'I', 'M'	dGJ
`pqH`	(p_H, q_H)	'H'(='J'), 'I'	dGH (redundant)
`Jbranch`	MBL branches	'S', 'K', 'R' (branches)	Individual branches
`ijd1`	(i_{d1}, j_{d1})	'S', 'K', or 'R'	dGd1
`ijS1`	(i_{S1}, j_{S1})	'S', 'K', or 'R'	dGS1
`ijp1`	(i_{p1}, j_{p1})	'H', 'I', 'M'	dGp1
`ijc1`	(i_{c1}, j_{c1})	'H', 'I'	dGc1 (redundant)
`p1branch`	MBL branches	'S', 'K', 'R' (branches)	Individual branches
(handles only needed for extended pseudoknots)			
`ijd2`	(i_{d2}, j_{d2})	'S', 'K', or 'R'	dGd2
`ijS2`	(i_{S2}, j_{S2})	'S', 'K', or 'R'	dGS2
`ijp2`	(i_{p2}, j_{p2})	'H', 'I', 'M'	dGp2
`ijc2`	(i_{c2}, j_{c2})	'H', 'I'	dGc2 (redundant)
`p2branch`	MBL branches	'S', 'K', 'R' (branches)	Individual branches

Supplement Table S2. List of the structure of the handles used in pseudoknot mapping: handle names, pointer contents (`pk_link`), tag contents (`pk_name`) and free energy values (`fPK`). The tags 'S', 'H', 'I' and 'M' were defined in Table S1. The tag 'K' indicates a core pseudoknot, 'R' an extended pseudoknot, and 'J' a junction (effectively identical to a hairpin loop 'H'). Core PKs ignore terms in the last part of the Table. MBLs are specified by the handles `Jbranch` (for junction), `p1branch` (root domain 1 at `ijp1`) and `p2branch` (root domain 2 at `ijp2`). The Free energy for the junction (dGJ) is evaluated at the closing point of the loop (p_l, q_l) and the correction to the free energy due to the presence of a pseudoknot is stored in dGwt (i.e., `pql`) as a weight. The set is certainly complete and even to some extent redundant in some cases.

Similar to the cPK, the ePK has an input (i_R) and output (j_R). However, instead of one root domain, there are two: domain 1 (i_{d1}, j_{d1}) (blue background region) and 2 (i_{d2}, j_{d2}) (purple background region). The linkage stem joins these two independent domains at the respective neck regions: (i_{p1}, j_{p1}) (neck region 1) and (i_{p2}, j_{p2}) (neck region 2). Special pointers in the handles map any structure in the loop (ss or PK). Similarly, the junction region also contains special handles to map out any complex structure contained in (p_J, q_J). Like the root domain of the cPK, the root domains of the ePK can consist of complex ss or even PKs. Hence, very complex structure can be mapped if some hierarchical folding pattern for a sequential 5' to 3' folding can be discerned.

More will be discussed in Section S2.3.2 on the diversity of PK structures that can be obtained. We only comment here that unlike other formal description schemes [23], the core and extended PK notation depends strongly on the way in which the RNA folds and the stability of the domains of RNA structure. The tag structure and mapping procedures depend on the order of the folding in this model. Therefore, these tags are not necessarily unique. However, they help describe the most likely pathway to the native state; something that is not offered by typical mapping methods. RNA is currently thought to fold according to the hierarchical folding hypothesis [25].

S2.2.3. Linkage stems

By employing a folding strategy to this problem, the linkage stem indicates something of the most likely order in which the PK structure formed. For typical ABAB PKs (simple cPKs), which stem is defined as the linkage stem is not particularly unique or significant. However, true linkage stems should be restricted to stems that are less than a full rotation (360°) of a contiguous A-RNA double helix (about 10 base pairs [26]) because all PK models exclude true knots. Proteins that have true knots have been observed [27]. Knots are quite possible and indeed should be common — as anyone who has ever untangled a "carefully wound" electrical cord can realize. Therefore, the length of a linkage stem on cPKs should be less than 10 contiguous bps; nevertheless, stems of this length appear to be extremely rare. There should be no other distinction than the most probable order of folding that is read into this information.

The linkage stem of an ePK reflects the initial domain structure that appeared before the linkage stem of the PK joined the independent RNA domains. Therefore, in ePKs, there is more significance behind what forms the basis of the linkage stem. Nevertheless, these definitions are often more qualitative and, thermodynamically, there is nothing unique about them.

S2.2.4. Look up tables of exit tags

There is one final issue with mapping. What happens if we build a PK and later add on another PK and have to edit the original PK slightly? This is a plausible situation. One way is to update the

internal object. However, given that the level of editing is not extreme, it is better to keep the original object unchanged because that represents the best structure over the region of the original PK. At present, the extent of editing appears to satisfy this condition. Hence, we have not constructed rules to handle drastic editing of the originally derived structure if it is embedded in a pseudoknot. Note that if the domain being edited is not a pseudoknot, then quite drastic editing is certainly allowed in vsfold5 even if pseudoknots are present within that same domain.

Therefore, instead of editing the original object, vsfold5 uses a lookup table of exit tags to decide if a region of structure should be evaluated. Any reconfiguring that would occur in a biological context would involve melting of material and reconfiguring the surroundings that were originally present in the original PK structure. This means we need to fix the original structure at the new neck region. This we can do by checking a short list of exit tags that indicate where some editing has occurred, these tell the methods to jump to the next task built up at the higher level.

S2.3. Time, memory and structural complexity

Here we describe the computational demands (time complexity) and memory requirements of vsfold5. In addition, we describe the structural complexity that is accessible to vsfold5.

S2.3.1. Time and memory complexity of vsfold5

Before discussing the time complexity, we must emphasize that there are numerous known inefficiencies built into the secondary structure methods of the current program because the focus has been on the science; not the algorithm. We expect more efficient code is likely to speed up the calculations significantly and reveal the true cost of the pseudoknot calculations. A benchmark test of the current program is shown in Table S3.

S2.3.1.1. Stems:

A major part of the time complexity in vsfold4 and 5 is devoted to finding a good combination of stems in the stem building methods. A full search should add a cost of $O(N^4)$ [28].

The stem search further includes recursive methods that scan different pathways to find the best effective stem length with the minimum free energy. Recursive scanning can extend up to 6 levels (default) and more can be requested, but six levels are generally sufficient for most problems. The search is done recursively until either a cutoff is reached or the stem clearly terminates. Because such recursive optimizations are done every time (even though past successful solutions are *already* known), it is currently a major design inefficiency. An ameliorating factor is that this search is limited to a finite number of recursive hops. Theoretically, reusing these solutions reduces the time complexity to as small as $O(N^3)$ [28]. However, because this program remains under extensive constant development, these optimizations are currently not used. Without any optimization, the time complexity appears to be $O(N^{4.7})$ for this calculation.

The memory requirements are essentially $O(pN^2)$, where p is the number of pointers (7: forward, reverse, free energy and tag Table S1).

S2.3.1.2. MBLs:

As with the case of the stem building modules, the MBL modules certainly add a time complexity of $O(N^3)$ [29]. The more complete analysis of the MBL's environment also adds considerable overhead.

Memory requirements also are increased with this approach due to the MBL branch pointers in general, and also the contents of the pseudoknot handles. However, the pointer size is of $O(N^2)$, and the maximum number of branches is an inverse function of the Kuhn length (a longer Kuhn length requires less allocation of memory for a sequence of length *N* because more structure is put

into building long stems and less is devoted to loops and branching). Therefore, the memory requirements are $O(pN^2)+O(mN^2)$, where p is the number of pointers (same as a stem) and m is the maximum number of branches (a number that is inversely dependent on the Kuhn length). The default value is 15 branches and therefore $30 \le m \ll N$ (for large *N*). Future development of an object that permits internal allocation of memory is expected to reduce this global demand for branch memory significantly.

S2.3.1.3. PKs:

Given that optimizations are done on the stem search methods that reduce the time complexity to the theoretical minimum of $O(N^3)$, the addition of PK capability will result in a time complexity ranging between $O(N^{3.5})$ and $O(N^4)$. This is comparable to some other recent methods [19]. A simple search for H-type PKs would normally be expected to cost $O(N^4)$ in time complexity. With this approach, the full structural complexity that normally requires $O(N^6)$ is not sacrificed; however, there are limitations introduced due to the choice of the folding pathway.

In the modeling, we assume that if a biologically active functional RNA requires a PK in the structure, the existing folded structure should leave “hints” that encourages the formation of the linkage stem. Though it is possible this assumption is sometimes inaccurate for particular examples, such counter examples appear to be few based on the current survey. Therefore, all free strand regions are checked in the search; however, only strands that already have at least 5 nts free are tested for potential PKs.

There are several reasons why this approach can permit full complexity without adding more than an order 1 increase in time complexity.

Mapping is able to reduce this to a fractional exponential increase. To show that mapping will reduce the time, we show the following thought experiment. In Fig. S12(1-8), stem loop structures are shown in increasingly complex patterns. The dominant surface where PK binding can happen is usually in the hairpins. (Vsfold5 does look everywhere for free strands, nevertheless, for the reason stated above it is often pointless.) Let s represent the average stem length and l represent the average loop length. Then with the given proviso, Figs. S12(1-8) should have the following total lengths: $N_1 \cong 2s+l$, $N_2 \cong 4s+l$, $N_3 \cong 6s+2l$, $N_4 \cong 8s+3l$, $N_5 \cong 10s+3l$, $N_6 \cong 12s+4l$, $N_7 \cong 14s+4l$, and $N_8 \cong 16s+4l$ respectively, where the subscript n in N_n indicates the number of stems in the structure. Of course, there are worst possible cases that could increase the content of l, but we think these examples are quite reasonable. This very roughly corresponds to $N_n \sim sn + l\sqrt{n}$. Then for large N, $n \sim N/s$. If we now consider the ratio of the loop surface relative to total length that the mapping methods search, we find that

$$\frac{l\sqrt{n}}{N} = \frac{l\sqrt{N/s}}{N} \propto \frac{1}{\sqrt{N}}. \qquad \text{(S0)}$$

Thus, the time complexity of the free strand search grows as $O(N^3 \times \sqrt{N})$ rather than $O(N^3 \times N)$. Furthermore, the mapping manages all forms of PKs with this growth because it scans for the linkage stem and then can quickly determine what type of structures are being linked. Hence, the solutions are not limited to H-type pseudoknots either.

In addition, the approach simply scans the free strand region on established domains or melts a hot lead onto them and re-computes the structure. These calculations are only carried out periodically; currently, the default is at the end of every third cycle of the secondary structure computation and internally within a MBL. Although linear, when combined with the above reductions, the time complexity is further reduced. Therefore, the PK-search option can result in as small an increase as $O(N^{3.5})$.

There are significant memory demands for PK handles (Tab. S3); however, this is not a large because only a few handles are actually made in any computation.

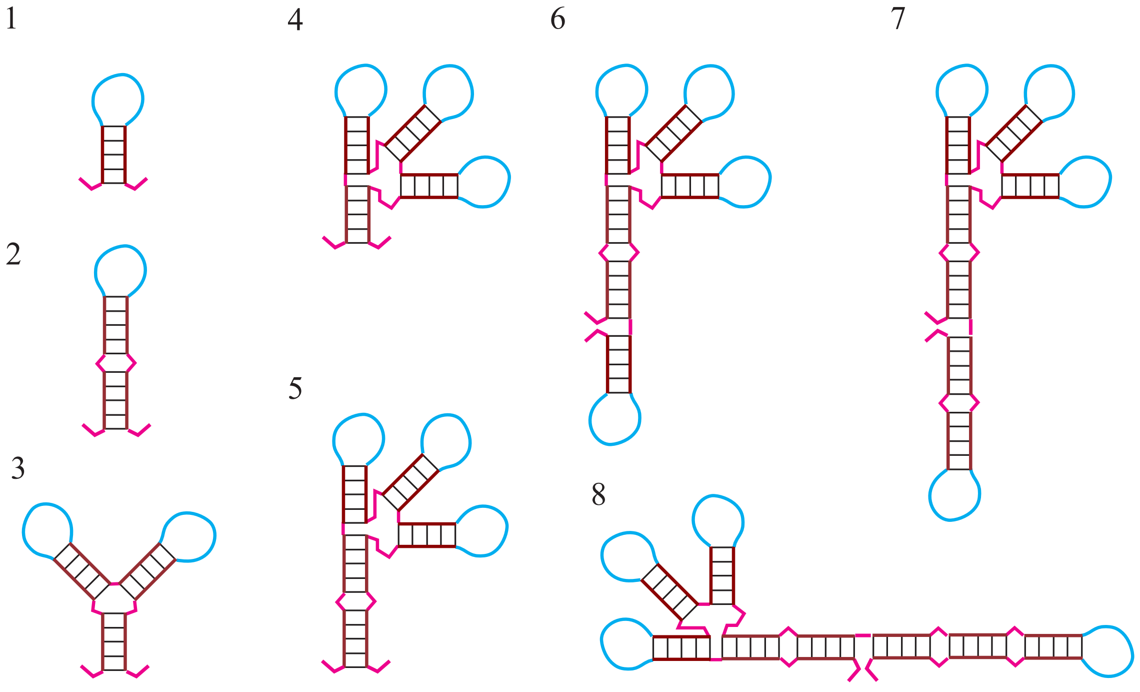

Supplement Figure S12. Examples of some possible hypothetical RNA structures with average stem lengths (s: brown) and loop free strand lengths (l: cyan): (1) single stem, (2) two stems plus a loop, (3) three stems, (4) four stems, (5) five stems, (6) six stems, (7) seven stems, and (8) eight stems. The magenta regions represent minor loops and free strand regions.

Sequence length [nt]	Vsfold4: observed calculation time [s]	Vsfold5: observed calculation time [s]
201	1.5	2.5
363	18	30
435	50	89
570	238	341
Exponent:	4.7	4.7
Time constant:	1.3×10^{-11} [s]	3.7×10^{-11} [s]

Supplement Table S3. A comparison of the computational time required for secondary structure (vsfold4) and pseudoknots (vsfold5). Here, N is the sequence length. (Calculated using Intel Xeon 3.0 GHz single processor, Red Hat Linux 9.2, gcc 3.3.)

S2.3.2. Structural complexity of pseudoknots of vsfold5

To this point, only the general mapping methods have been described. Here we consider some examples of how these simple motifs can be used to build some of the complex structures important to state of the art approaches.

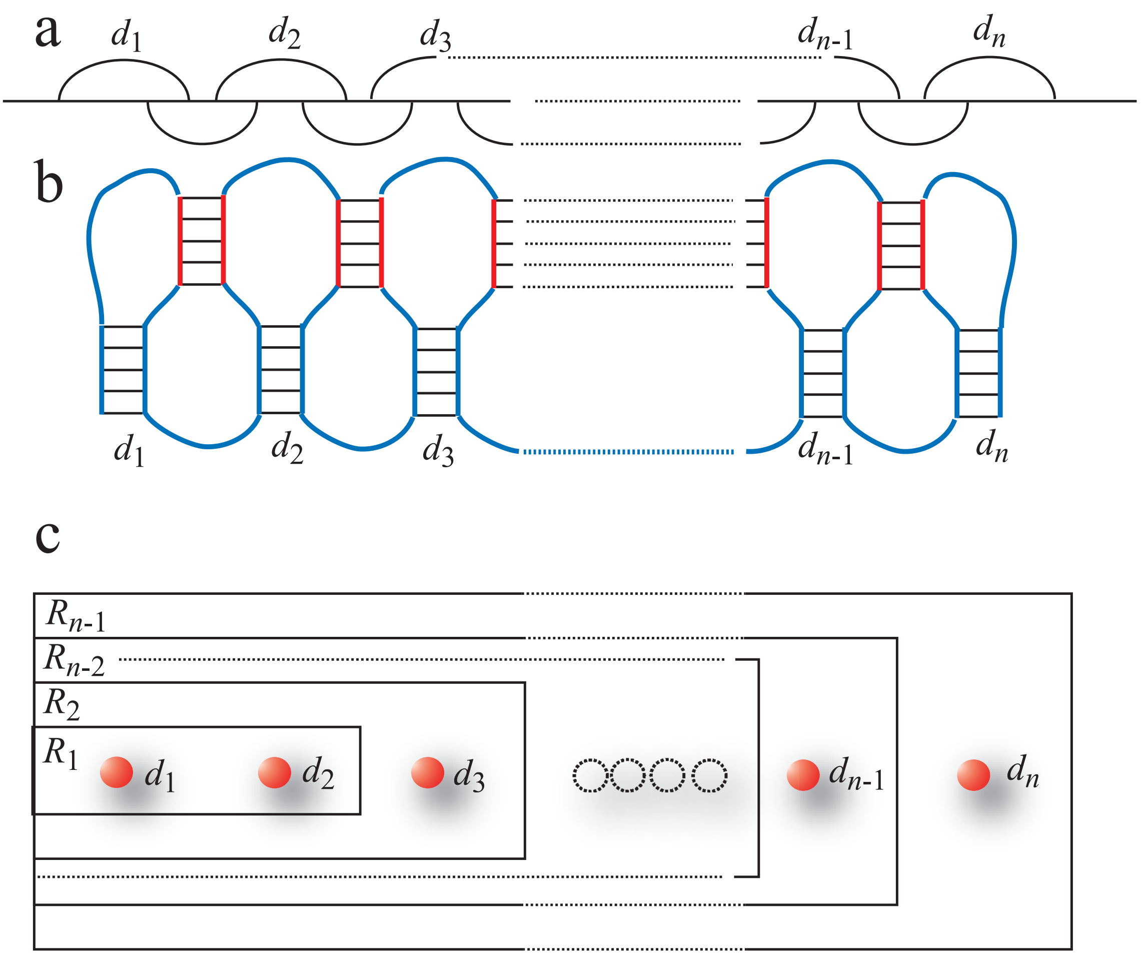

Supplement Figure S13. An example of a recursive extended pseudoknot. (a) the Rivas and Eddy style Feynman diagram [18] of a recursive structure of extended pseudoknots. (b) The corresponding secondary structure with pseudoknots. (c) The object representation of this pattern in which the assignment of an ePK takes the form of a Russian doll.

S2.3.2.1. Recursive pseudoknots

With the two basic structures (cPKs and ePKs), recursive PKs can be built [30]. In Figure S13, we show an example of a recursive ePK that works successively along a single pattern. In Figure S13a, the Rivas and Eddy Feynman diagram [18] of a recursive ePK and below this in Fig. S13b, the

corresponding structure is shown in succession. Below this, in Fig. S13c, a simple example of the folding construction is shown in which each domain (d_1, d_2, etc.) is made and a linkage made to the previous member in a regular progressive pattern.

In Figure S13c, the dot indicates an individual domain (also labeled above as d_n in Fig. S13b). These are surrounded by a box that is labeled R_k that indicates the successive assignment tags for the ePK domains. The box enclosing the dots indicates the objects inside. Therefore, the label R_1 contains d_1 and d_2, the label R_2 contains the objects R_1 and d_3, etc. The object structure resembles a Russian doll in form where each time one opens the box, a smaller doll appears.

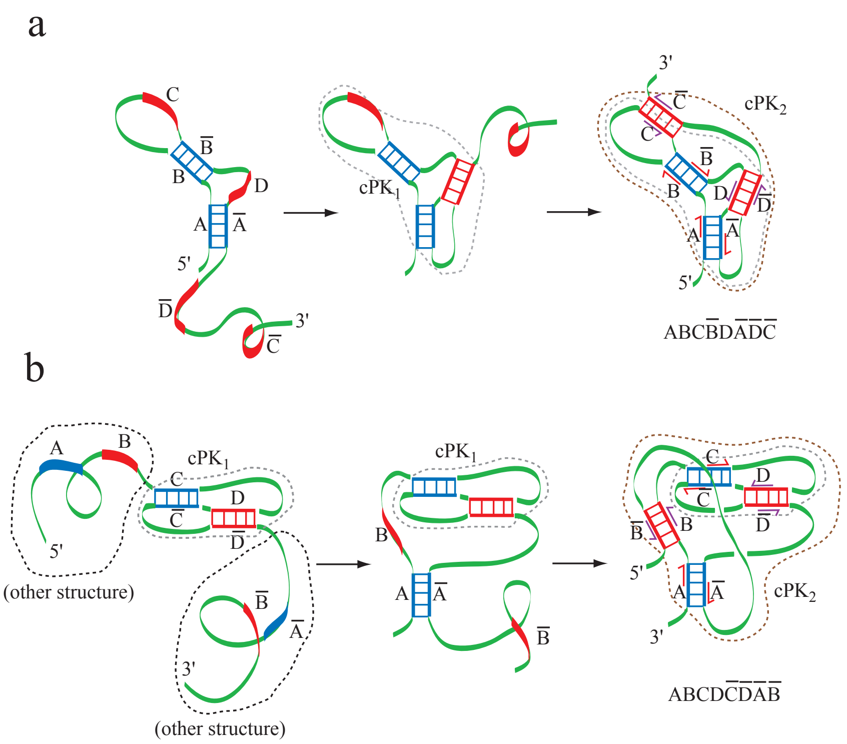

Supplement Figure S14. Examples of some ways to map complex pseudoknots as a series of cPK motifs derived from a suggested folding pathway. (a) An example of a recursive cPK with the pattern $\mathrm{ABC\bar{B}D\bar{A}\bar{D}\bar{C}}$. (b) An example of a group of embedded cPKs with the pattern $\mathrm{ABCD\bar{C}\bar{D}\bar{A}\bar{B}}$. The arrow suggests a dominant folding pathway that the structures could form in a sequential folding (5’ to 3’) progression.

The arrangement of the boxes need not be in such a trivial order as shown in this example. However, once a PK is decided from some folding pathway, this wrapping shields the internal contents inside (though this aspect could be modified too). Any recursive pattern of construction of this kind requires unwrapping the box before the contents inside can be explored. Hence, it is not

essential that it follows exactly the plan of this example, but, currently, some patterns could be missed if the priority of construction is disordered relative to the sequential folding direction.

This process is done procedurally with recursive construction methods, but resembles that of wrapping the objects inside.

Although Fig. S13 is an example done with ePKs, it is also possible for such a pattern to form with cPKs. This again is reflecting something of a view of how RNA structure is formed. The pattern generated would be identical, but the process by which it came about is not. With the ePK, a pair of blue stems would form first and these would then fuse together. With a cPK, linkage would be added successively to the structure. Generally, in this model, a stand-alone linkage stem requires independent stability of whatever internal structure is inside. On the other hand, an extended PK depends on the stability of its independent domains (for example, d_1 and d_2) and their mutual coupling together to form a small linkage stem. Far more complex mixtures of structure are likely to be built up with an ePK.

As a second set of examples, we consider how a pseudoknot with the pattern $\mathrm{ABC\bar{B}D\bar{A}\bar{D}\bar{C}}$ (Fig. S14a) and the pattern $\mathrm{ABCD\bar{C}\bar{D}\bar{A}\bar{B}}$ (Fig. S14b) would form [24], where, for readability, we have added a bar over certain letters to indicate the complimentary strand of the corresponding letter: *e.g.*, A and $\mathrm{\bar{A}}$. The folding pathway is shown in Fig. S14. Figure S14a shows a recursive PK formed by adding more stem onto an existing PK. Figure S14b shows a cPK embedded inside another cPK. Hence, many types of complex PKs can be found with this strategy.

S2.3.2.2. Limits on structural complexity:

With some limitations specified here, vsfold5 can map any structure that can be expressed by some reasonable sequential (5' to 3') folding scenario using these simple cPK and ePK motifs. The concepts behind vsfold5 permit a capacity to find all different types of pseudoknots with minor restrictions that we lay out here.

1. Pseudoknot stems should be significant in size. Vsfold5 rejects isolated 1, 2 and 3 base pair (bp) linkage stems because there is simply insufficient information to assess such tiny interactions with the raw estimates available from the thermodynamics. That the current estimates work at all is a credit to the power of thermodynamics to forgive gross ignorance and hardly an indication that the values used are precise.
2. A variable Kuhn length is not currently available on vsfold5 yet. Therefore, a PK of variable flexibility is not necessarily accessible due to the level of maturity of the current approach.
3. For any complex pathway architecture of PKs, there must be at least one pathway that satisfies the folding conditions: $5' \rightarrow 3'$ folding, denature/refolding conditions, or $3' \rightarrow 5'$ folding. Currently, vsfold5 only processes $5' \rightarrow 3'$ folding, but, in principle and with some further

development, its modularity would admit denature/refolding or $3' \rightarrow 5'$ folding. Currently, for structural RNA, this does not appear to be problematical. Nevertheless, some structures could be excluded for this reason.

4. Pseudoknots currently do not check themselves in vsfold5. Therefore, whereas a recursive chain of extended PKs could form (Fig. S13), connecting the ends, as in Fig. S15d, is currently not done. Nevertheless, this example *can* be done, if one really wishes and in this respect, vsfold5 can do better than even the best available approach currently.
5. Finally, the PK structure does require that hierarchical folding occurs with RNA. Since the base pairing free energies (FE) are of similar order of magnitude for AU, GC, and GU pairing, this is probably true. However, we cannot say for certain at this point that a pathologically variable free energy surface of extreme difference in FE is universally solvable by any method other than an exhaustive search, particularly if the configurations are also arbitrarily unrestricted. Nevertheless, if such a surface can be found, all the current strategies would suffer the same defeat.

With this general proviso, all the structures indicated in the historic developments in this field are doable in this approach; given some consistent folding pathway is describable.

The program can solve structures such as those shown in Fig. S15. Structures (c) and (d) were considered in Ref. [30] and structure (e) is discussed in Ref. [24]. Labels on the Rivas and Eddy Feynman diagrams [18] in Fig. S15 indicate the stem sequence and its compliment (e.g., A and $\bar{A}$). The structures in Figs. S15a-c can all be evaluated with vsfold5 in its current form.

Exceptions, as pointed out in item 4, are such structures as Fig. S15d (first pointed out in Ref. [30]) and S15e (pointed out in Ref. [24]).

For Figure S15d [30], only the loop around (d_1 and d_n) is not possible in the current vsfold5 architecture. Presently, vsfold5 does not look inside of its own pseudoknots for more pseudoknots. It is assumed that pseudoknots build up modularly inside of one another, if they build up at all in this way, and we need not concern ourselves with modules deep inside the superstructure until we need to unwrap them. Moreover, the types of structures likely to satisfy even this are small because of steric hindrance of jamming two stems in a loop and folding such a structure. This would only be more problematical for joining d_1 and d_n (Fig. S13) together. Perhaps with sufficient length, a circle could be made that would satisfy all the physical requirements. To handle this prediction problem, we would need to add exit tags for domains in the lookup table and apply them in the same way as the neck-exit-tags. Currently, aside from the aspect of theoretical curiosity, such matters, though in principle doable, have not been opened in the current architecture of the program.

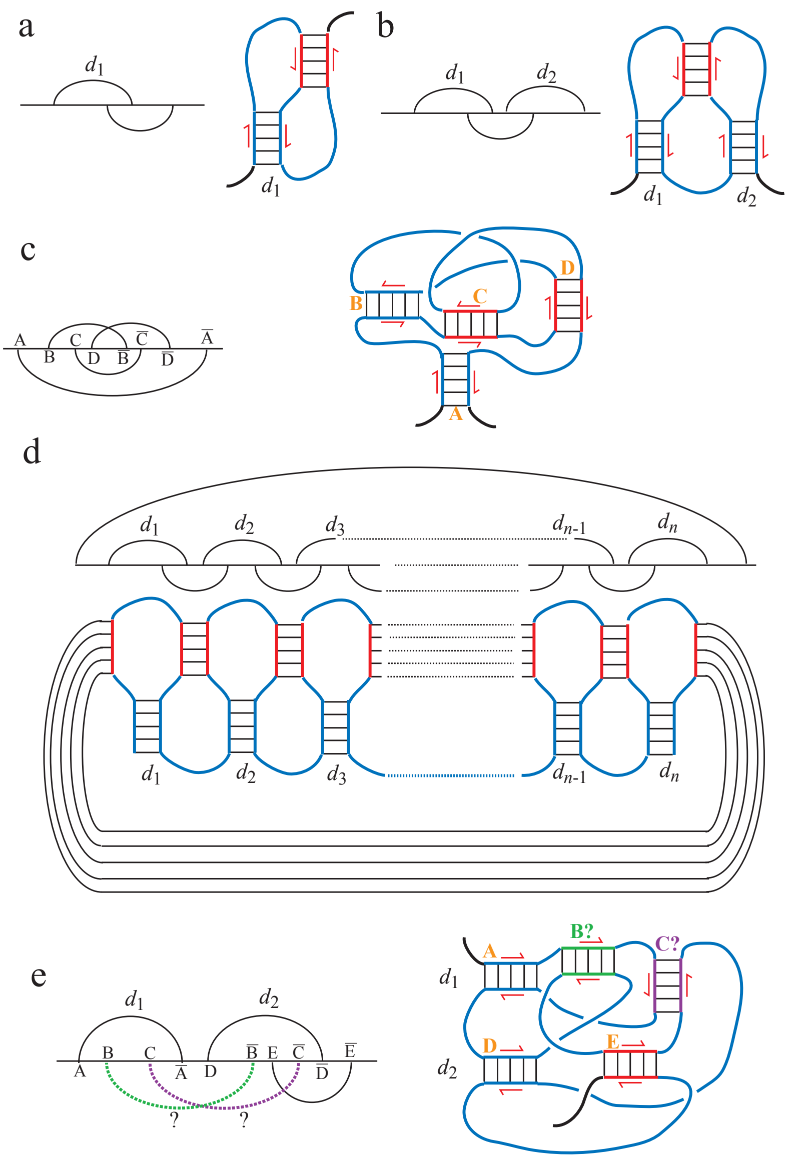

Supplement Figure S15. Rivas and Eddy type Feynman diagrams of RNA structure and the corresponding structure represented in secondary structure style. (a) A core pseudoknot. (b) An extended pseudoknot. (c) a consecutive set of core pseudoknots embedded in a piece of secondary structure. (d) A recursive pseudoknot that folds back on itself. (e) A complex interplay of an extended PK and a core PK. Vsfold5 cannot currently examine its own pseudoknots. Therefore, in example (d), the loop around cannot be computed, and, in example (e), either stem B or stem C can be obtained, but not both.

For Figure S15e, for the pattern $ABC\overline{A}D\overline{B}E\overline{C}\overline{D}\overline{E}$ [24], currently vsfold5 can chose either B or C, but not both from the general pathway of folding domain 1 and 2 first. Estimating from the general tendency of the free energy rules, stem C is probably more likely. In short, we don't currently ask PKs to examine themselves. Nevertheless, there is an alternative pathway shown in Fig. S16 that can succeed with this structure. Hence, the order is a crucial factor in this model. Even this can be remedied with little additional cost in time complexity, were this deemed critical in prediction problems.

Hence, there is in fact no sacrifice in computational possibility at all and, indeed, vsfold5 may have even more capability than any existing approach. However, there are some additional restrictions on what is admissible due to the current state of extreme uncertainties and perhaps some (largely correctable) idiosyncratic assumptions made in the design and current development of the program.

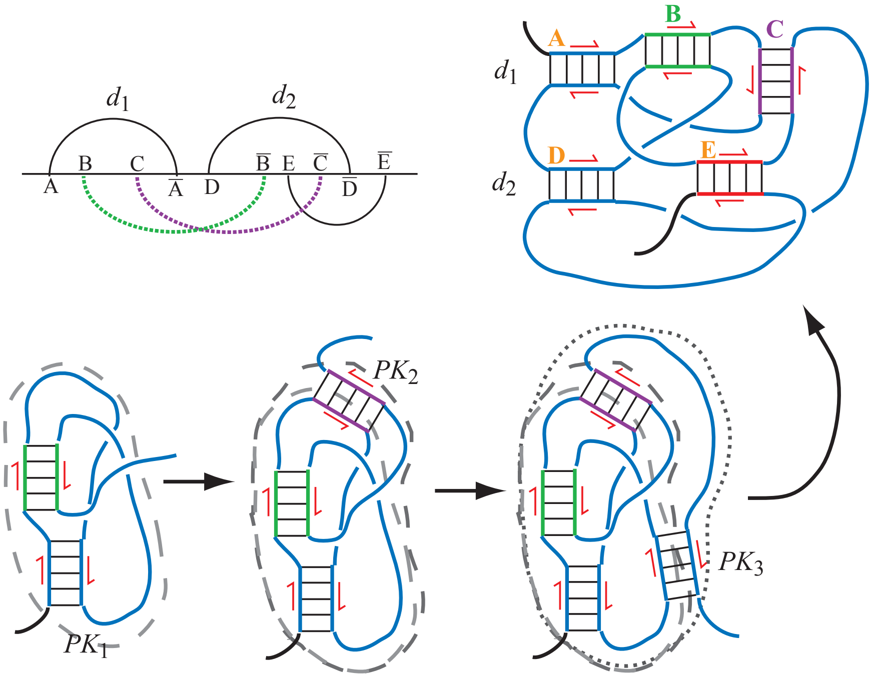

Supplement Figure S16. An alternative pathway that leads to the exact structure listed in Fig. S15e.

S2.4. Free energy evaluation of pseudoknot structures

Here we explain the general concepts behind the evaluation methods of pseudoknots. Most of the secondary structure methods are done as before and can be found in the literature [1].

The free energy is path independent in the CLE model. Therefore, the choice of linkage stem has only the indication of the most likely pathway for the structure to fold and carries no other significant information beyond this.

There are a multitude of structural issues associated with pseudoknots that can often be ignored when the problem only involves secondary structure. The reason appears to be due largely to the fact that the space between different secondary structures is sufficiently large that sub-domains are not as likely to interfere with each other. Not so for the pseudoknot. Though RNA bases all exhibit a similar size and chemical identity, packing issues are important particularly due to electrostatic effects. Hence, PKs begin to show some of the same difficulties encountered in protein structure problems where both packing and residue characteristics in some context can make or break the structure prediction [31] once the PK problem is introduced.

Currently, the methods presented here are largely based on qualitative estimates and are subject to considerable revision in the future.

S2.4.1. Helical twist of RNA

In all cases tried so far, the linkage stems tend to have less than 10 contiguous bps in a single stem; a situation where a knot would form [26,32]. It is often the case that the important linkage stems only contain 4 to 6 bps (close to the 180° twist) and, often, when they do exceed 5 bps, they have an internal loop that breaks the continuity at 5 bps. This renders the issue about the twist largely moot. Generally, RNA appears to use internal loops (I-loops) to help compensate for the twist, basically defeating most attempts to use these relations in some general way. A cutoff of 9 contiguous bps on the linkage stem is easily added.

S2.4.2. Local contact strain in very short loops:

If the loop accepting a linkage is far too small, then there is very little flexibility in the loop to accommodate sufficient twist between the loop and the linkage stem. We refer to this here as local contact strain.

This is expressed graphically in Fig. S17a and in 3D in Fig. S17b. Several problems emerge, particularly when considering Fig. S17b. First the free strand (orange) connecting the linkage stem (red) is rather short to accommodate the large rigid structure that is being forced on the space. Worse is that the root stem (blue) is filled up with the linkage stem. Neither can the root domain make up the difference because it is already bending under the strain of a tight loop. Second, the loop regions

on both halves are short, so there is no flexible free play between the forming strands. Over 5 bps, the twist in the stem is roughly 180°, and when this must be coupled with such a short loop, there is no room to accommodate either part well. Finally, nucleic acids have negatively charged phosphates that must be compensated by divalent cations in tight configurations, yet there is hardly any surface area where such divalent cations can rest long enough to have much impact on the stability.

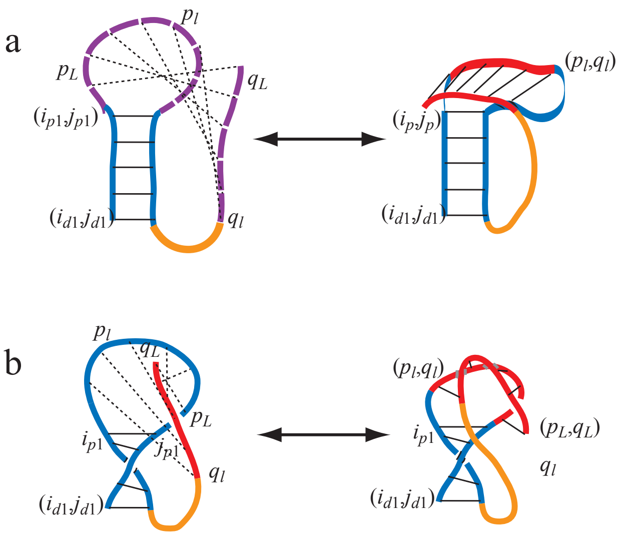

Supplement Figure S17. An example of the concept of "local contact strain": shown diagrammatically in (a) and 3 dimensionally in (b) with the structure on the left representing the condition prior to adding a PK, and on the right, representing the condition after adding a PK.   The orange strand indicates the junction region, red the linkage and blue the root domain (purple indicates the region being joined). Over the range of 5 bps, the double strand helix of A-RNA rotates approximately 180°. This renders the distortions and lack of free play even more severe in (b) than would be suggested by the diagrammatic example in (a).

When too much of the stem is occupied by the linkage, the part occupied by the stem restricts the free motion of the loop, changing the flexibility and distorting the structure of the loop. Therefore, the costs of formation should increase when too much of the loop is occupied by the linkage stem. This contact strain tends to select root domains with a larger free strand contact region for PK formation compared to tight loops. When the free strand within the root domain is less than 2 nt, the structure is strongly discouraged unless melting some of the existing stem can achieve a visible improvement. The persistence length, or more precisely when referring to a Gaussian polymer chain, the "Kuhn length" (ξ), is also important to consider here because the stiff structure should only increase resistance to tight structures such as this.

Given a stem of length l_J (measured in the number of bases) and a loop of length n_l, the available free strand is $s_f = n_l - l_J$. Currently, there is a minimum strand length for this gap ($s_{\mathrm{F}}^{\min}$) such that

$$dG_{gap} = \begin{cases} 3.2 k_{\mathrm{B}} T, & s_{\mathrm{F}}^{\min} \geq n_l - l_J \\ 0, & s_{\mathrm{F}}^{\min} < n_l - l_J \end{cases} \quad \text{(S1)}$$

where $s_{\mathrm{F}}^{\min}$ is also scaled as a function of the Kuhn length (ξ) such that $s_{\mathrm{F}}^{\min} = \xi / 2$. (The Kuhn length is a measure of the stiffness; hence, the Kuhn length will tend to stiffen the strands.) There are known loops that have only 1 nt free, and to this, the above cost will be added. Experimentally, at least some of the PKs that have such a short strand also require Mg^{2+} to form. Therefore, this does not seem an unreasonable cost to add to very short loops.

Very short PK structures ($j_R - i_R \leq 25$ nt), as found with some frame shift related pseudoknots [33,34], require special attention to address. In line with the observations of Huang *et al.* [4], at least some discrimination can be worked out from the specific features of the PK. We do not analyze to the same detail as Huang *et al.* However, stem lengths are evaluated similarly with at least one stem requiring equal free strand length or the above cost is applied. Sequence composition and coaxial stacking may also be a factor in the stability of these structures but is currently not considered.

For cPKs, formation costs come from both the contact strain (local in character) and standard stem stability issues that govern all secondary structure stem formation rules. There is no natural distinction between linkage stems and the root domain, so all the basic rules of secondary structure must be applied in the same way to all stems.

For ePKs, coaxial stacking and parallel stem interactions can help stabilize the linkage stem, hence, standard stem stability issues are relaxed slightly for ePKs. A minimum size for an ePK is also applied because two independent domains need sufficient contact region to warrant formation of a linkage between them. When the loop is short, considerable distortion is needed to accommodate the linkage stem. Again, a heavy cost for building a stem on small a loop region is added when $s_{\mathrm{F}}^{\min} \geq n_l - l_p$ occurs in Eqn (S1). Moreover, when $j_R - i_R \leq 25$ nt, the two independent domains are so small that formation of such a structure is essentially impossible to satisfy even were the Kuhn length equal to 3 nt (a ridiculously short length for most structured RNA).

S2.4.3. Pleating as a factor in reducing strain on a pseudoknot:

An important feature is pleating and reorientation of the superstructure. For example, the group I intron of *Tetrahymena thermophila* has a stem that is folded over at the J5/4 junction position of the structure [35,36]. Hence, this effect should be considered when an internal loop is sufficiently large; at least for more than 3 nt × 3 nt (3×3).

When pleating occurs, the stem can no longer resemble anything close to a straight stick-like object, but is folded back on itself. If, for example, we have a single pleat (as in the *Tetrahymena thermophila* example at the junction J5/4), then the effective length becomes the length of the P4 stem minus the length of the P5 and P5abc stems or $l_p = l_{\mathrm{P4}} - l_{\mathrm{P5+P5abc}}$. In principle, this permits calculating an effective length (l_p) for any number of pleats

$$l_p = \sum_{k=1}^{n} (-1)^{k-1} l_k \qquad \text{(S2)}$$

where k is the index of a stem corresponding to l_k (when there is only one stem $k=1$ and $k>1$ means there are $k-1$ pleats).

Currently, there is no information available on the FE of pleating and consequently the parallel stem-alignment interactions between the P4 and P5 stems are unknown (though probably attractive). Therefore, the current algorithm does not ascribe any gain or loss from this effect; rather it simply evaluates a plausible effective length for the stem and adds a small entropic cost of $1.5k_{\mathrm{B}}T$ for each pleat, where we currently assume the cost is electrostatic effects in the junction. We currently assess stems with internal loops greater than 3×3 as "plausible". Only the plausible effective length is considered and if even this cannot be satisfied, then surely the PK structure cannot form. An example of pleating is shown diagrammatically in the main text (Fig. 4d).

S2.4.4. Mutually disproportionate lengths

In addition to contact strain, both cPKs and ePKs must be able to combine in an energetically favorable way with each other. In developing the concepts here, we have adapted the concept of flexible loops and rigid rods first attempted successfully in Isambert *et al.* (Refs. [9] and [26]). The importance of considering the structure of the PK in a more 3D perspective is worth emphasizing, and in this respect, the elaborate architectures worked out in the approach of Ref. [9] are an important direction to take in considering PKs. The advantage of this approach is that the method of rejection is based on a slightly more objective approach of thermodynamics rather than a somewhat subjective approach of predefined elimination of certain structure types.

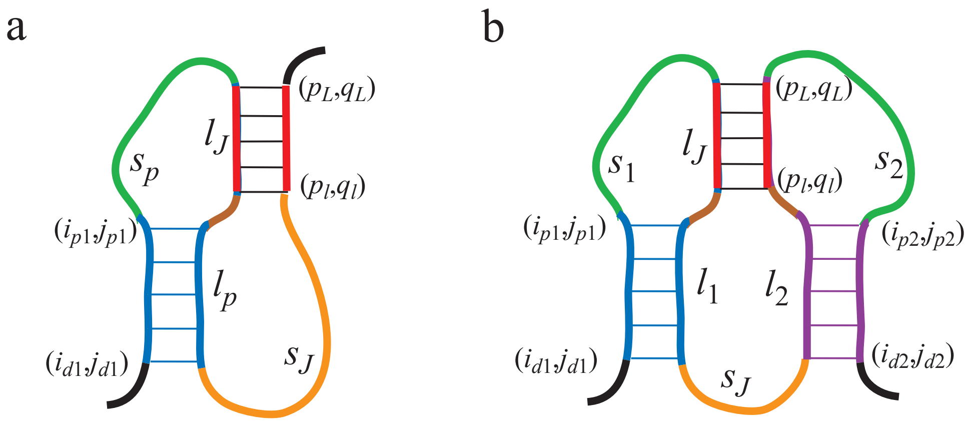

Supplement Figure S18. A schematic of the strands that comprise the basic pseudoknots. (a) A core pseudoknot. The brown indicates the region shared by the root stem loop and the linkage stem loop. Blue indicates l_p (the root stem), green&brown s_p, orange s_J and red l_J (the linkage). (b) An extended pseudoknot. The brown indicates the region shared by the root stem loops and the linkage stem loop. Blue indicates l_1 (root stem 1), purple l_2 (root stem 2), green&brown s_1 (or s_2), orange s_J and red l_J (the linkage).

Disproportionate lengths for cPKs:

We begin with a simple example and gradually develop the concept from this kernel.

Figure S18a shows a schematic of a simple H-type cPK. Because the schematic is not shown in 3D, it suggests that s_J must stretch out a length l_p to be able to form the linkage stem at l_J. In fact, there is a helical twist (Section S2.4.1) that can ameliorate this and there are differences between the minor grove and the major grove (Section S2.4.2 and Ref. [4]). Ideally, these factors should be considered.

However, beyond these trivial examples, a general way to envision a 3D structure of arbitrary complexity becomes rather unwieldy without any experimental information to support it. Currently, because of this paucity of experimental information, vsfold5 handles the problem in the rather simple-minded way diagrammed out in Fig. S18a for all cPK structures. This ensures that there is no overt bias introduced by the foreknowledge of a precise structure for small cPKs and utter ignorance for large cPKs.

As a result, the stretching of s_J by an amount l_p (Fig. S18a) is assumed in these calculations, where l_p is the effective length of the root domain (Section S2.4.3). Similarly, s_J is also evaluated in terms of its effective length such that secondary structure, which shortens the actual stem length in the junction region, is taken properly into account. We considered including

corrections for the twist [26,32] but found it was unnecessary at this level of maturity — though obviously important in principle to keep in mind. Though we sacrifice some accuracy, the structures are treated uniformly this way. When more experimental data is available, a far more precise module can be constructed.

Using the general form of the CLE model, we estimate the initial length of the coil r_i in the free strand s_J region (Fig. S18a) is (Ref. [1])

$$r_i^2 \sim \xi s_J b^2 \tag{S3}$$

where s_J describes the number of nt in the junction sequence, b is the distance between the nucleotide residues in the chain (units of length) and ξ is the Kuhn length (units nt). Because the stem is a rigid rod, it tends to be relatively straight, and therefore, the final length after folding (r_f) transforms to

$$r_f \sim 0.5b(l_p)^{0.5+\varsigma} \tag{S4}$$

where l_p is the effective length of the stem (possibly shorter than the apparent stem due to the pleating effect; Section S2.4.3), the factor of 0.5 estimates the relative contour length of the stem (about 3 Å in A-RNA [26]) and ς is a stretching parameter on the coil length: $0 \le \varsigma \le 1/2$.

Let $l = l_p$, then the entropic contribution from stretching s_J against l_p is

$$\begin{aligned}
-T\Delta S &= -T\left(\Delta S(r_f) - \Delta S(r_i)\right) \\
&= -k_{\mathrm{B}}T\left\{\gamma \ln\left(r_f^2 / r_i^2\right) - (\gamma + 1/2)\left[\left(\frac{r_f}{\bar{r}}\right)^2 - \left(\frac{r_i}{\bar{r}}\right)^2\right]\right\}\left(\frac{l_J}{\xi}\right) \\
&= k_{\mathrm{B}}T\left\{\gamma \ln\left(2s_J / l^{1+2\varsigma}\right) - (\gamma + 1/2)\left[1 - \left(l^{1+2\varsigma}/(2s_J)\right)\right]\right\}\left(l_J / \xi\right)
\end{aligned} \tag{S5}$$

where we have used the definition of the coil state to write $\bar{r} = r_i$ and γ is the self avoiding weight (discussed in Ref. [1]). For the simple Gaussian model, $\gamma = 1$. Currently, vsfold5 uses the standard weight used in traditional approaches ($\gamma = 1.75$) as the default. To this expression, as with other renormalization examples [1], a weight of the linkage stem (l_J) divided by the Kuhn length is included: l_J / ξ corresponding to l_J contributions spread over a length scale ξ nt. In more complex RNA structures, the length s_J is the effective free strand length. The entropy contribution to the FE in Eqn (S5) depends on the ratio l_p / s_J .

In most examples, $s_J \le l$ and therefore, the second term of Eqn (S5), surrounded by square brackets, will contribute a large stretching effect. Hence, for cPKs, some strands would not be able

to connect if the entropic free energy contribution from stretching s_J is too large.

The thermodynamics is blind to the definitions of linkage and root stems because the folding is path independent. To insure that there is no bias in evaluating the free energy, Eqn (S5) is computed by exchanging labels (l_p with l_J and s_J with s_p) in Fig. S18a and doing the analysis in the same way. The resulting free energy values are then added. This way, whichever way the structure folded, the final result is independent. It also reflects the property of equal and opposite reaction and response.

We herein emphasize that this approach is quite preliminary and is likely to be revised substantially in the future when more precise 3D information can be worked directly into the code.

The concept is similar to Ref. [26]. The difference is that the full equation for the entropy change is used rather than just the stretching part (the right hand side of Eqn (S5)) and this equation is applied both ways: exchanging root and linkage stems. In addition, the weight parameter does not assume a Gaussian model ($\gamma = 1$) and adjusts to match γ to the value used in all other calculations (default $\gamma = 1.75$). Currently, $\delta = 2$ (see Ref. [1]) because there is no information about level of correlation between the stems in the structure. This latter point too can be easily remedied to handle a variable δ and Flory parameters.

Disproportionate lengths for ePKs:

For the ePK, we can apply a similar approach (in Fig. S18b). However, unlike the cPK where s_J is the only element between l_p and the linkage stem l_J, the stem l_2 lies between l_1 and l_J. Therefore, we must consider the difference in the relative lengths of stems l_1 and l_2 (as well as s_J) in accounting for the FE. The difference in the length of the parameter l in Eqn (S5) is closer to $l = l_1 - l_2$. Hence, we write $l = |l_1 - l_2|$ and evaluate Eqn (S5) as before.

Currently, the weight for ς in Eqn (S4) is set to 0 for cPKs and 0.5 for ePKs. The reason for the difference is that the free strand on the cPK has more ways to accommodate the length differences by flexible rearrangements than the equivalent problem with the ePKs, particularly when large domains are involved. In both cases, strong deviations are strongly discouraged when the stems are drastically different in length and cannot be compensated reasonably using pleating.

S2.4.5. Orientation (insufficient compensating free strand) strain:

So far, we have only considered the case where the relationship between the effective stem length of the root domain and the adjoining linkage stem could be placed in direct relation to each other. However, the relative positioning of the linkage stem on the root domain (or domains) is also important.

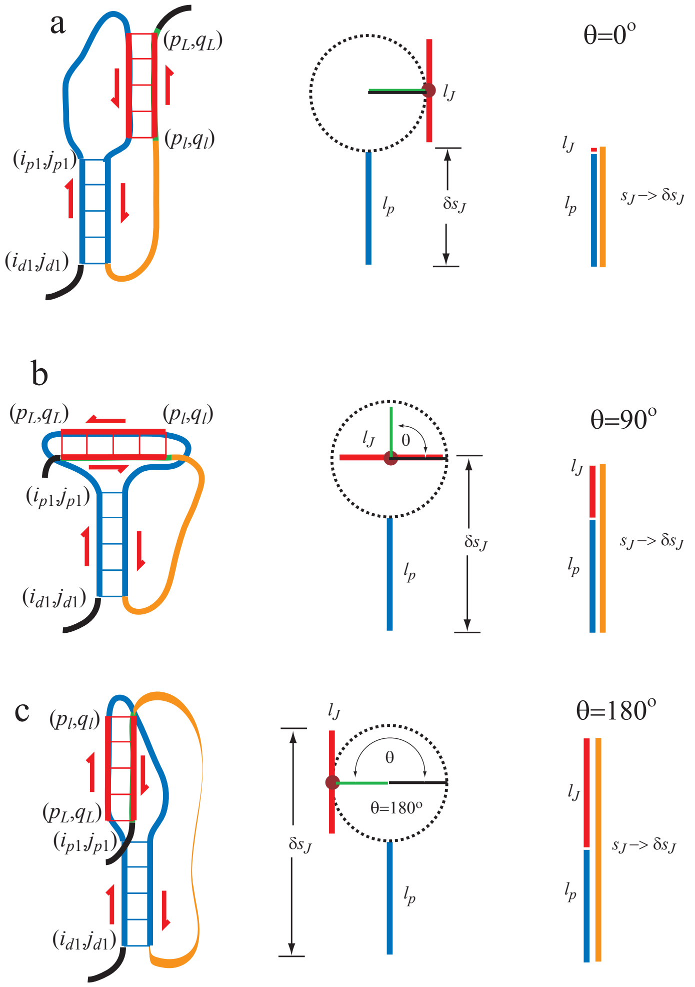

Supplement Figure S19. An example of the concept of orientation strain for a core PK. Here, different angles θ are expressed using a graphical representation, a simple angle diagram and finally a stick diagram of the estimated lengths. (a) For $\theta = 0$ rads, the angle and length scale is the smallest. (b) For $\theta = \pi / 2$ rads, the angle and length scale is has increased by half of the linkage stem (l_J). (c) For $\theta = \pi / 2$, the angle is the largest and equal to the sum of the lengths l_J and l_p (the root stem).

Disproportionate lengths for cPKs:

Figure S19 shows three angle relationships for the linkage stem of a core PK. When the linkage stem is angled at π/2 rads on the loop, some additional length approaching 1/2 the linkage stem (l_J) is needed. When $\theta = \pi$ rads, it suggests that the length is approaching the sum of both the root stem (l_p) and the linkage stem: $l = l_p + l_J$. This suggests the following minimal angle relationship

$$l^2 = l_p^2 + l_J^2 \left(1 - \cos\theta\right)/2 \tag{S6}$$

where l is used in the same way as in Eqn (S5).

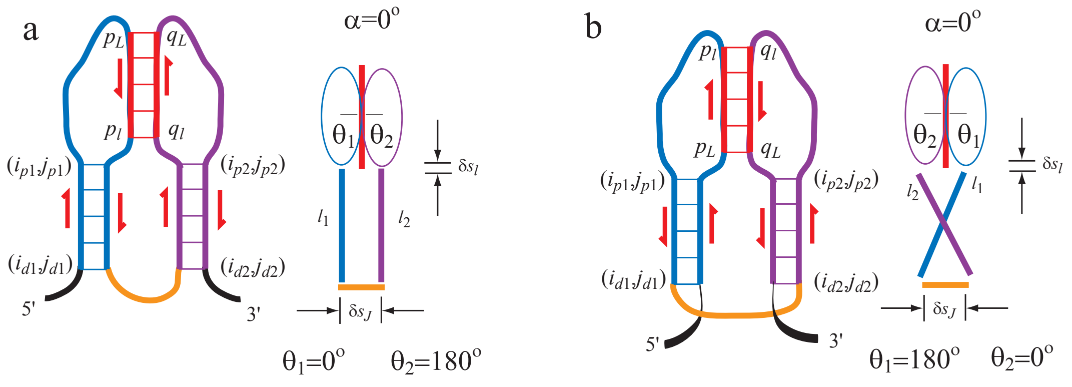

Supplement Figure S20. A model for the phase angle for the ePK where l_1 and l_2 are equal but have opposite phase angles. (a, left) An example of the standard structure where $\theta_1 = 0$ and $\theta_2 = \pi$ rads. (a, right) A simplified representation of this diagram. (b, left) The same example with the phase rotated by π radians: $\theta_1 = \pi$ and $\theta_2 = 0$ rads. (b, right) A simplified representation of this diagram. The colors correspond to those shown in Figs. S18 and S19: (cyan) domain 1, (purple) domain 2, (orange) junction region and (red) linkage stem.

Disproportionate lengths for ePKs:

Extended PKs require additional considerations because the structure involves two domains of effective stems. In Figure S20, the relation between the two stem-loops is such that their lengths (l_1 and l_2) are equal and their relative phase (α) is zero. The first example (Fig. S20a) is the standard for that this structure is typically drawn in: $\theta_1 = 0$ and $\theta_2 = \pi$ rads. The second example (Fig. S20b) has the angles in the loops rotated by an angle π rad: $\theta_1 = \pi$ and $\theta_2 = 0$ rads. There is more symmetry and therefore more degeneracy in the structural orientations of the domains in an ePK.

Figure S21 shows what happens when we move away from this very simple example. What appears to matter more is the difference in the orientation of the angles or the phase of the angle. Figure S20a is the same in terms of angles with Fig. S21a. However, the phase angle on Fig. S21b is certainly π/2 rad and Fig. S21c π rad. More free strand is needed to accommodate this increased length in the stem. The relationship for l_1 and l_2 suggests the following approximation

$$l^2 = l_1^2 + l_2^2 - 2l_1 l_2 \cos\left(\alpha(\theta_1, \theta_2)\right),\ \alpha = \left|\theta_1 + \theta_2 - \pi\right|. \qquad \text{(S7)}$$

Thus, for $\alpha(0,\pi) = \alpha(\pi,0)$, $l = l_1 - l_2$ (Fig. S21a), and for $\alpha(\pi,\pi)$, $l = l_1 + l_2$ (Fig. S21c). For $\alpha(\pi/2,\pi)$, $l^2 = l_1^2 + l_2^2$ (Fig. S21b).

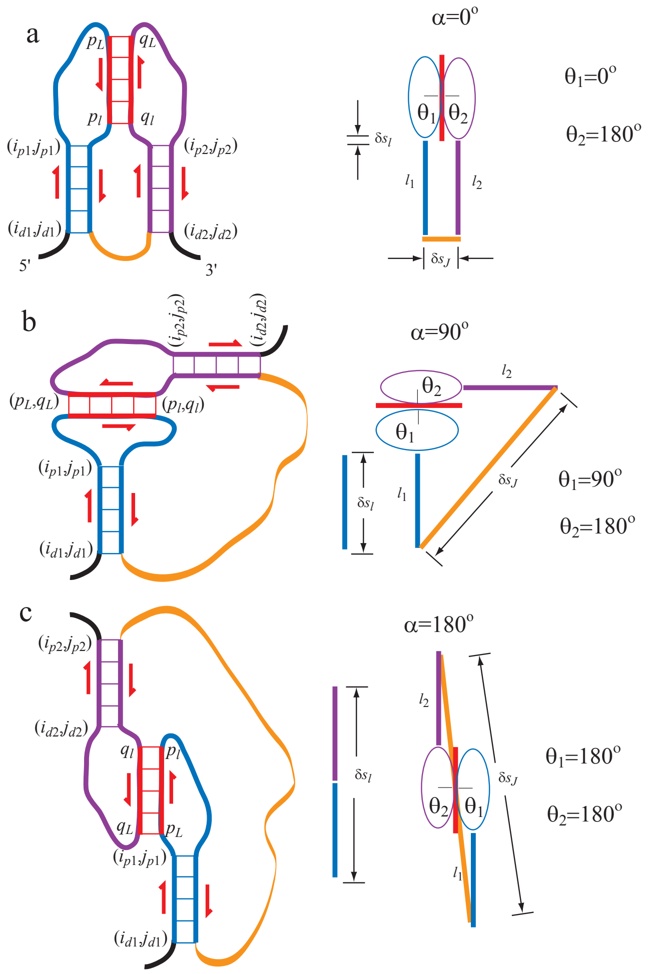

Supplement Figure S21. Examples of orientation strain in various ePK configurations. Here, the orientation strain follows the same pattern as the cPKs but is more pronounced because of the added length of the second root domain. On the left hand side, a graphical representation of the different angles θ_1 and θ_2. In the middle is a stick representation of the distances. On the right hand side is the same cartoon with a stick and angle representation. (a) $\theta_1 = 0$ and $\theta_2 = \pi$ rads. (b) $\theta_1 = \pi/2$ and $\theta_2 = \pi$ rads. (c) $\theta_1 = \pi$ and $\theta_2 = \pi$ rads.

The calculation of this cost is carried out for both domains to account for the different orientations, thus there is no differentiation between different structural configurations and the results are always independent of the order of combination. The formation only depends on whether the thermodynamics find a favorable free energy for the given structure.

These effects are partly ameliorated by using effective stem lengths for l_1 and l_2. In general, when the complex domains interact, their relationships are essential because the larger part of the structure is compact and cannot drastically change shape.

When secondary structure or other pseudoknot units are added to these simple schematics shown in Fig. S18, the length of the free strand available for cutting slack in the length is reduced.

Currently, the algorithm's heuristics assume that there is no change in these subdomains after formation. Therefore, these structures are assumed frozen in form when the length of free strand is evaluated. However, even were we to open up some of the sub-domains located in the junction and root domain regions, the algorithm can evaluate the resulting structure according to a systematic rule that is completely reversible.

In general, examination of the detailed processes going on within sequential folding and the search information we examined, there is little to be gained by ripping up already formed structure to hook on an emaciated linkage stem. It is more likely that evolution has already committed a lot of the effort needed to prepare a sequence for the best folding pathway. There is the possibility that intermediate protective structure may have been selected by evolution. However, even when we do see some indications of a stable intermediate stem in the location of a linkage point, there is still some surface where a "hot-lead" at the 3' end can fuse onto the root domain structure. The gain between the "before" and the "after" should be significant. Finally, if a linkage stem is really supposed to extend a domain, evolution is likely to select for configurations that make that linkage stem interface easy to find.

Hence, random sequences and folding in non-biological contexts where the vector direction of the sequential folding is ignored may result in very different predictions than the current algorithm's heuristics handle, but evolution (with 3.5 billion years behind it) has surely selected sequences that avoid many of these major pitfalls.

S2.4.6. Coaxial stacking, parallel stem alignments and steric repulsive effects:

A final important category involves the arrangement of stems within the super structure: coaxial stacking, parallel stem alignments, and neighboring linkage stems (Figs. S22 and S23).

S2.4.6.1. *Coaxial stacking:*

An example of coaxial stacking is shown in Fig. S22a. Currently, vsfold5 cannot compute any gains from coaxial stacking, only a small reduction in entropy loss for some simple cases. Moreover, the

coaxial stacking is only computed when it is a proximal stem within the secondary structure or proximal to a linkage stem. Future plans are to add modules using the global mapping strategy to evaluate complex architectures of coaxial stacking.

S2.4.6.2. *Parallel alignment of stems:*

An example of parallel alignment of stems is shown in Fig. S22b. The RNA tends to have some level of mutual attraction. Hence, there should be considerable stabilization when stems can be arranged in parallel [37]. In addition to coaxial stacking, pseudoknots appear to require far more consideration of parallel stem alignment. Pleating is another example of simple parallel stem alignment where stabilization is likely to result from the mutual self attraction of the RNA. Currently, there is scant information on these type of interactions. As a result, these contributions are currently ignored. However, future plans are to develop modules to address stem arrangements.

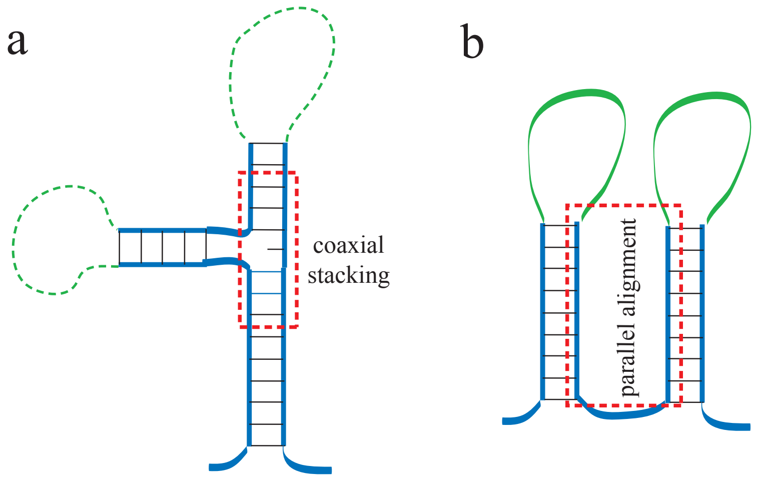

Supplement Figure S22. Examples of coaxial stacking and parallel alignment between two stems. Coaxial means that both stems are along the same axis. Parallel alignment means that the stems are positioned in parallel.

S2.4.6.3. *tandem pseudoknot stems:*

The last group of interactions that are currently considered is the situation of tandem linkage stems (Fig. S23). In a pseudoknot calculation, it can often happen that some large domain has a linkage stem that can, in principle, associate with another domain where a linkage stem neighbors it. However, RNA has physical dimensions (Fig. S23c). Currently, we exclude these linkages if they result from independent domains if the existing linkage (Fig. S23c: PK1) and the new linkage (Fig. S23c: PK2) in the developing ePK border at a distance less than 4 nt away. There is the possibility that there could be coaxial stacking between such domains, but currently, there is no evidence, and

such matters require more data to make a definitive decision about how to model the effect properly.

Other types of tandem linkages such as those shown in Figs. S23a and b are allowed.

In the case of Fig. S23a, the linkages are actually located on independent domains and may even have some coaxial stacking occurring between them. Examples of this situation are found with the tobacco mosaic virus where there are three tandem PKs (see Fig. 1e of the main text). In such a case, there is no true distinction between a linkage stem and the root domain. Hence, it would not be correct to exclude such structures.

In the case of Fig. S23b, the linkages are on the same root domain and are immediately tandem to one another. Again, there is no true physical distinction, as the linkage stems and the root stem can be easily exchanged.

We *expect* that it is necessary to develop more sophisticated considerations, but currently, these general categories appear to be sufficient to discriminate between valid structures and invalid ones (at least for the structures so far tested). It would be desirable to develop some coaxial stacking models for special cases such as that shown in Fig. S23a. More experimental data is needed to understand these structures well before the model can be improved significantly.

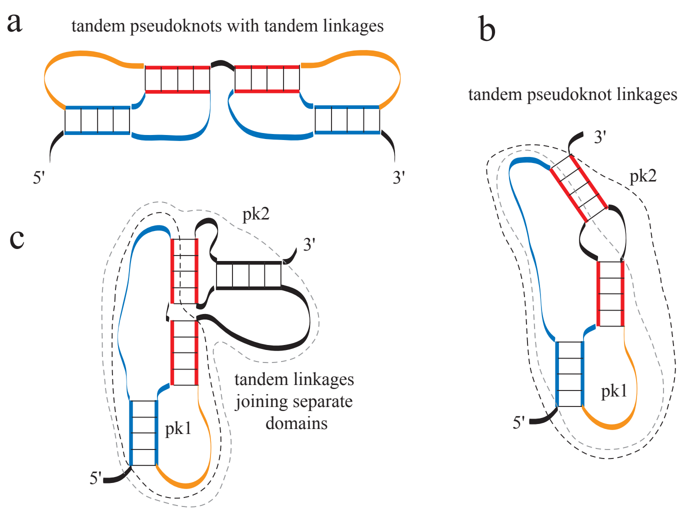

Supplement Figure S23. Examples of different types of tandem linkage stems. (a) The linkage stems are right next to each other, but on independent domains. (b) The tandem linkages that are along the same axis and form a single pseudoknot, but are wrapped by the folding order. (c) The tandem linkages are formed by joining two independent domains and forming a extended PK. Examples (a) and (b) are allowed, (c) is not.

S2.4.7. General folding methodology

In the search for PKs, the heuristics first looks for contacts that are significant in magnitude to warrant further testing. If a potential contact is found where $dG < -2$ kcal/mol, a stem melting module will try to melt part of the structure in the root domain to see if better contact can be achieved. Occasionally, this delivers a true edit. However, because the free energy of stems are of similar order of magnitude, most of the time, it appears that no gain is achieved.

At the same time these local stem-melting methods are testing the minor rearrangements around the neck of the root domain and the linkage stem, the heuristics also continually build up a structure starting at the 5' end and moving progressively toward the 3' end searching for the best structure. While the main structure is building up, a "leading edge" of sequence ranging from 7 nt to 20 nt ahead of the currently built up structure (also called the dwell region) is tested on this existing structure for possible pseudoknots. This we refer to as a "hot lead" because the process resembles melting the leading edge strand onto the existing structure. When the folding catches up with this already tested structure, the free energy built by the secondary structure construction methods at the same region is then compared with the free energy generated by the pseudoknot test. If the pseudoknot turns out to be more stable than the structure generated by the secondary structure construction methods, then the PK is selected. This latter effect is generally far more drastic and appears to be the most effective.

A 3D structure will also be affected by entanglement issues [8,9]. However, vsfold5 only considers the thermodynamically most-probably folding pathway in the calculation. Entangled structures represent alternative and less favorable structures. Hence, the entanglement issues are completely circumvented. Therefore, although a genuine issue, we need not monitor these or assign any cost function for them; nor should we, unless we intentionally wish to build them. Moreover, whatever cost could be derived would have to take into account base triples and probably other tertiary structure interactions. Since there is virtually no context dependent thermodynamic data for base triples, it is difficult to make more than a qualitative estimate of the stability of such structures at the present time.

S2.4.8. Future directions in structure prediction using vsfold

Thermodynamics and statistical mechanics are important in every physical process studied in science and engineering. Yet thermodynamics remains one of the poorest approaches to structure prediction of RNA and proteins; particularly when sequences become very long. Here, we explain some points where we observe that the predictions appear to improve within the current model. Whether these improvements are sufficient to overcome these broader issues remains to be seen.

First, as structures grow larger, the 3D features of the structure and their interactions with other parts of the structure become very important. Stems can form coaxial stacking. Stems can also

condense into complex parallel interaction arrangements. These are common features of the RNase P (Ref. [38]) and the group I intron (Ref. [35]) that were tested in this work. The folding of these structures into modules of tightly packed, interacting structures requires context dependent experimental parameters that are currently rare or non-existent.

The current 3D evaluation methods in vsfold5 are of the most trivial and simple-minded of models. These models appear to be sufficient for simple pseudoknots; however, they lack enough generality to handle the far more complex structural interactions that can develop in large compactly folded RNA sequences: particularly molecules like RNase P [38]. Certainly, we should expect that the mapping information of vsfold5 can be exploited to consider these compact 3D structural interactions; particularly if this is combined with experimental data that directly measures the thermodynamics of these interactions. Presently, even for the few 3D structural motifs that vsfold5 detects in a calculation, these contributions are ignored because we don't know what objective parameters to assign to them.

Notably, the authors in Refs. [7] and [9] have also tried to look at 3D structural issues in their models; some of it quite detailed. Considerable experimental information is needed to significantly improve and test our model as well as these other models.

Second, though it was computationally expedient to use a single Kuhn length for vsfold5, the flexibility of a real RNA structure (an inverse function of the Kuhn length) is likely to vary; particularly in most long functional RNA sequences. Furthermore, the flexibility can be context dependent and different stages of the life cycle of an active RNA can lead to changes in structure. For example, this is known to occur in the life cycle of HIV-1 [39]. Likewise, nascent RNA is surely different from well annealed RNA [40]. More experimental data is needed to understand the environmental conditions that influence the Kuhn length. No clear information is currently available.

Predictions would certainly be helped if the Kuhn length was adjustable to appropriate context dependent values. Even a simple adjustment at the level of a domain would help (see Refs. [41-43] for these definitions). For example, we found that we could obtain the correct structure between P9 and P9.2 in Fig. 2c (main text) by simply cutting the sequence at P6 and using a smaller Kuhn length to fit the sequence from P6 to P9.2 (Ref. [1]). Therefore, varying the Kuhn length is an approach that shows measurable promise. This would certainly help biologists if all that is required is to cut up the domains and look at the closer details.

We are currently developing an automated method for selecting the Kuhn length (ξ). However, more information is needed about the relationship between the experimental conditions and the Kuhn length. Further study of real RNA should be expected to require some critical thinking on the part of the user.

Third, proteins are often involved with larger structures. There is not a lot we can do about that until we have a model to understand protein-RNA interactions. Vsfold4 and 5 have aimed at

understanding what the RNA does in the absence of these proteins. Therefore, prediction failure with Vsfold4 and 5 may indeed be informative because it can help us understand the contribution from protein interactions.

Finally, we cannot emphasize enough the importance of including partition function information and suboptimal structure into these calculations. In this work, we have shown that directional folding (5' to 3') may be significant in RNA folding problems. Refolding experiments reflect different experimental conditions to the natural course of folding. Nevertheless, trapped intermediates are observed (and should be expected) regardless of the direction of folding. They are, at best, only amplified in refolding experiments. A partition function calculation tells us the distribution of states. We have shown that the distribution of similar structures is sorted far better using the same strategy used by vsfold4 and 5 (see Refs. [41,42]). That study included rRNA (1534 nt). From these studies, we saw not only that we could obtain a better selection of the right structure from the solution set; we saw that all similar structures tended to group together with that structure. This also means that other dominant suboptimal structures can also be detected because they tend to appear in groups. This is what we should expect of a distribution of structures in a partition function and what we indeed observed. No other approach has ever demonstrated this capability. Therefore, to study the states of a riboswitch, this approach offers some advantage in suboptimal structure detection.

The major advantage that vsfold5 offers is that the calculation is stable. By this we mean that when the domain size exceeds a certain limit (a function of the Kuhn length), there is no further change in the predicted structure [41,42]. This does not mean that vsfold5 picks the correct structures in that domain; rather, beyond a certain length, it does not corrupt whatever structure was predicted. In general, this means that a user who selects a poor (5' to 3') sequence fragment to study and a poor Kuhn length can still obtain some useful information from vsfold5; though the utility of the calculation is often greatly diminished. This stability is a feature that no other approach can claim. Therefore, we have managed to address part of the major issue raised in Ref. [22]. Because we have achieved stability, we can now begin to address the other major problems.

References

1. Dawson W, Fujiwara K, Futamura Y, Yamamoto K, Kawai G (2006) A method for finding optimal RNA secondary structures using a new entropy model (vsfold). Nucleotides, Nucleosides, and Nucl Acids 25: 171-189.
2. Martinez HM (1990) Detecting pseudoknots and other local base-pairing structures in RNA sequences. Methods in Enzymology 183: 306-318.
3. Chen J-H, Le S-Y, Maizel JV (1992) A procedure for RNA pseudoknot prediction. Comp Appl In Biol Sci 8: 243-248.
4. Huang X-L, Ali H (2007) High sensitivity RNA pseudoknot prediction. Nucl Acids Res 35: 656-663.
5. Ren J-H, Rastegara B, Condon A, Hoos HH (2005) HotKnots: heuristic prediction of RNA secondary structures including pseudoknots. RNA 11: 1494-1504.
6. Van Batenburg FHD, Gultyaev AP, and Pleij CWA (1995) An APL-programmed genetic algorithm for the prediction of RNA secondary structure. J Theo Biol 174: 269-280.
7. Cao S, Chen S-J (2006) Predicting RNA pseudoknot folding thermodynamics. Nucl Acids Res 34: 2634-2652.
8. Xayaphoummine A, Bucher T., Thalmann F, Isambert H (2003) Prediction and statistics of pseudoknots in RNA structures using exactly clustered stochastic simulations. Proc Natl Acad Sci U S A 100: 15310-15314.
9. Xayaphoummine A, Bucher T, Isambert H (2005) Kinefold web server for RNA/DNA folding path and structure prediction including pseudoknots and knots. Nucl Acids Res 33: W605-610.
10. Tabaska J, Cary R, Gabow H, Stormo G (1998) An RNA folding method capable of identifying pseudoknots and base triples. Bioinformatics 14: 691-699.
11. Liu H, Xu D, Shao J-L, Wang Y-F (2006) An RNA folding algorithm including pseudoknots based on dynamic weighted matching. Comp Biol and Chem 30: 72-76.
12. Raun J-H, Stormo GD, Zhang W-X (2004) An iterated loop matching approach to the prediction of RNA secondary structures with pseudoknots. Bioinformatics 20: 58-66.
13. Matsui H, Sato K, Sakakibara Y (2005) Pair stochastic tree adjoining grammars for aligning and predicting pseudoknot RNA structures. Bioinformatics 21: 2611-2617.
14. Uemura Y, Hasegawa A, Kobayashi S, Yokomori T (1999) Tree adjoining grammars for RNA structure prediction. Theor Comp Sci 210: 277-303.
15. Rivas E, Eddy SR (2000) The language of RNA: a formal grammar that includes pseudoknots. Bioinformatics 16: 334-340.
16. Akutsu T (2000) DP algorithms for RNA secondary structure prediction with pseudoknots. Discrete Appl Math 104: 45-62.

17. Huang C-H, Lu C-L, Chiu H-T (2005) A heuristic approach for detecting RNA H-type pseudoknots. Bioinformatics 21: 3501-3508.
18. Rivas E, Eddy SR (1999) A dynamic programming algorithm for RNA structure prediction including pseudoknots. J Mol Biol 285: 2053-2068.
19. Reeder J, Giegerich R (2004) Design, implementation and evaluation of a practical pseudoknot folding algorithm based on thermodynamics. BMC Bioinformatics 5: 104.
20. McCaskill JS (1990) The equilibrium partition function and base pair probabilities for RNA secondary structure. Biopolymers 29: 1105-1119.
21. Dirks RM, Pierce NA (2004) An algorithm for computing nucleic acid base-pairing probabilities including pseudoknots. J Comput Chem 25: 1295-1304.
22. Doshi KJ, Cannone JJ, Cobaugh CW, Gutell RR (2004) Evaluation of the suitability of free-energy minimization using nearest-neighbor energy parameters for RNA secondary structure prediction. BMC Bioinformatics 5: 105.
23. Pasquali S, Gan HH, Schlick T (2005) Modular RNA architecture revealed by computational analysis of existing pseudoknots and ribosomal RNAs. Nucl Acids Res 33: 1384-1398.
24. Condon A, Davy B, Rastegari B, Tarrant F, Zhao S (2004) Classifying RNA Pseudoknotted Structures. Theor Comput Sci 320: 35-50.
25. Tinoco I, Bustamante C (1999) How RNA folds. J Mol Biol 293: 271-81.
26. Isambert H, Siggia ED (2000) Modeling RNA folding paths with pseudoknots: application to hepatitis delta virus ribozyme. Proc Natl Acad Sci U S A 97: 6515-6520.
27. Virnau P, Mirny LA, and Kardar M (2006) Intricate knots in proteins: function and evolution. PLoS Comp Biol 2: e122.
28. Zuker M (2000) Calculating nucleic acid secondary structure. Curr Opin Struct Biol 10: 303-310.
29. Zuker M, Stiegler P (1981) Optimal computer folding of large RNA sequences using thermodynamics and auxiliary information. Nuc Acids Res 9: 133-148.
30. Lyngso RB, Pedersen NS (2000) RNA pseudoknot prediction and energy-based models. J Comp Biol 7: 409-427.
31. Levitt M, Gerstein M, Huang E, Subbiah S, Tsai J (1997) Protein folding: the endgame. Annu Rev Biochem 66: 549-79.
32. Aalberts DP, Hodos NO (2005) Asymmetry in RNA pseudoknots: observation and theory. Nuc Acids Res 33: 2210-2214.
33. Kolk MH, van der Graaf M, Wijmenga SS, Pleij CW, Heus HA, *et al.* (1998) NMR structure of a classical pseudoknot: interplay of single- and double-stranded RNA. Science 280: 434-438.
34. Felden B, Florentz C, Giege R, Westhof E (1996) A central pseudoknotted three-way junction imposes tRNA-like mimicry and the orientation of three 5' upstream pseudoknots in the 3'

terminus of tobacco mosaic virus RNA. RNA 2: 201-212.

35. Cate JH, Hanna RL, Doudna JA (1997) A magnesium ion core at the heart of a ribozyme domain. Nat Struct Biol 4: 553-8.
36. Woodson SA (2005) Structure and assembly of group I introns. Curr Opin Struct Biol 15: 324-330.
37. Lescoute A, Westhof E (2006) The interaction networks of structured RNAs. Nucl Acids Res 34: 6587-6604.
38. Torres-Larios A, Swinger KK, Krasilnikov AS, Pan T, Mondragon A (2005) Crystal structure of the RNA component of bacterial ribonuclease P. Nature 437: 584-587.
39. Berkhout B, van Wamel JL (2000) The leader of the HIV-1 RNA genome forms a compactly folded tertiary structure. RNA 6: 282-295.
40. Paillart JC, Dettenhofer M, Yu X-F, Ehresmann C, Ehresmann B, Marquet R (2004) First snapshots of the HIV-1 RNA structure in infected cells and in virions. J Biol Chem 279: 48397-48403.
41. Dawson W, Suzuki K, Yamamoto K (2001) A physical origin for functional domain structure in nucleic acids as evidenced by cross-linking entropy: part I. J Theo Biol 213: 359-86.
42. Dawson W, Suzuki K, Yamamoto K (2001) A physical origin for functional domain structure in nucleic acids as evidenced by cross-linking entropy: part II. J Theo Biol 213: 387-412.
43. Dawson W, Kawai G, and Yamamoto K (2005) Modeling the long range entropy of biopolymers: A focus on protein structure prediction and folding. Recent Res Devel. Experimental & Theoretical Biol 1: 57-92. (ISBN: 81-7895-167-3)
